# Targeting tumor cell-to-macrophage communication by blocking Vtn-C1qbp interaction inhibits tumor progression via enhancing macrophage phagocytosis

**DOI:** 10.7150/thno.94537

**Published:** 2024-04-22

**Authors:** Chen Zhang, Yi Liu, Jiayu Jiang, Chong Chen, Zhaojun Duan, Huifang Su, Shijian Wang, Baorui Tian, Yi Shi, Rong Xiang, Yunping Luo

**Affiliations:** 1 The School of Medicine, College of Pharmacy, Nankai University, Tianjin 300071, China.; 2 Department of Immunology, Institute of Basic Medical Sciences, Chinese Academy of Medical Sciences; School of Basic Medicine, Peking Union Medical College, Beijing 100005, China.; 3 Changzhou Xitaihu Institute for Frontier Technology of Cell Therapy, Changzhou 213149, China.

**Keywords:** Breast cancer, CRISPR screen, Vtn-C1qbp, macrophage, phagocytosis

## Abstract

**Background:** Cancer cells are capable of evading clearance by macrophages through overexpression of anti-phagocytic surface proteins known as "don't eat me" signals. Monoclonal antibodies that antagonize the "don't-eat-me" signaling in macrophages and tumor cells by targeting phagocytic checkpoints have shown therapeutic promises in several cancer types. However, studies on the responses to these drugs have revealed the existence of other unknown "don't eat me" signals. Moreover, identification of key molecules and interactions regulating macrophage phagocytosis is required for tumor therapy.

**Methods:** CRISPR screen was used to identify genes that impede macrophage phagocytosis. To explore the function of Vtn and C1qbp in phagocytosis, knockdown and subsequent functional experiments were conducted. Flow cytometry were performed to explore the phagocytosis rate, polarization of macrophage, and immune microenvironment of mouse tumor. To explore the underlying molecular mechanisms, RNA sequencing, immunoprecipitation, mass spectrometry, and immunofluorescence were conducted. Then, in vivo experiments in mouse models were conducted to explore the probability of Vtn knockdown combined with anti-CD47 therapy in breast cancer. Single-cell sequencing data from the Gene Expression Omnibus from The Cancer Genome Atlas database were analyzed.

**Results:** We performed a genome-wide CRISPR screen to identify genes that impede macrophage phagocytosis, followed by analysis of cell-to-cell interaction databases. We identified a ligand-receptor pair of Vitronectin (Vtn) and complement C1Q binding protein (C1qbp) in tumor cells or macrophages, respectively. We demonstrated tumor cell-secreted Vtn interacts with C1qbp localized on the cell surface of tumor-associated macrophages, inhibiting phagocytosis of tumor cells and shifting macrophages towards the M2-like subtype in the tumor microenvironment. Mechanistically, the Vtn-C1qbp axis facilitated FcγRIIIA/CD16-induced Shp1 recruitment, which reduced the phosphorylation of Syk. Furthermore, the combination of Vtn knockdown and anti-CD47 antibody effectively enhanced phagocytosis and infiltration of macrophages, resulting in a reduction of tumor growth in vivo.

**Conclusions:** This work has revealed that the Vtn-C1qbp axis is a new anti-phagocytic signal in tumors, and targeting Vtn and its interaction with C1qbp may sensitize cancer to immunotherapy, providing a new molecular target for the treatment of triple-negative breast cancer.

## Introduction

Phagocytosis by macrophages plays an important role in tumor control 1. It is a multi-step process involving target cell recognition, engulfment, and lysosomal degradation, which is regulated by ligand-receptor interactions, also known as phagocytosis checkpoints, between target cells and phagocytes 2. Since the identification of the CD47-SIRPα axis as the first phagocytosis checkpoint, other phagocytosis checkpoints involved in evasion of tumor cells from phagocytic clearance have been discovered, including the PD-1-PD-L1 axis, the MHC-I-LILRB1 axis and CD24-Siglec-10 axis 3-6. Tumor cells can evade clearance by macrophages through upregulation of these "don't eat me" signals. Monoclonal antibodies antagonizing the interaction of CD47 with its receptor SIRPα can eliminate tumor cells in vitro and in vivo and have shown therapeutic potential in several cancers 7. However, as CD47 is ubiquitously expressed, potential problems with using anti-CD47 antibodies as anti-cancer therapy include possible off-target effects such as anemia 8. Additionally, a portion of cancer patients do not respond to CD47-targeting therapy. Meanwhile, studies on the magnitude and persistence of the response to these drugs have revealed that there are other unknown "don't eat me" signals. Therefore, the discovery of these potential phagocytosis checkpoints is critical.

CRISPR screens have been employed to identify key genes required for macrophage phagocytosis of different substrates, including purified myelin, yeast (yeast cell wall particles), and sheep erythrocytes 9. There have also been studies exploring key genes intrinsic to the resistance of tumor cells to antibody-mediated phagocytosis 10. However, the phagocytosis of tumor cells by macrophages is mainly governed by ligand-receptor pairs between macrophages and tumor cells, and these ligand-receptor pairs have been reported rarely. In the current study, to find the phagocytosis-regulating ligand-receptor pairs existing in tumor cells and macrophages respectively, we performed a CRISPR screen using an in vitro FASC-based phagocytosis assay with direct phagocytic capacity as readout. By sorting out macrophages that had engulfed tumor cells by flow cytometry, and then mapping the genes that impeded macrophage phagocytosis in macrophages and tumor cells in cell-cell interaction database, we identified the ligand-receptor pair of Vtn-C1qbp.

Vitronectin is a multifunctional glycoprotein found in blood and extracellular matrix that can bind to a broad range of ligands and play a role in mediating protein hydrolysis, participating in immune responses and cell attachment, spreading, and migration 11-14.

C1qbp was originally isolated as a receptor for the globular head of the C1q molecule 15. It has been reported that Vtn can bind to the mature form of C1qbp, but barely to the C1qbp lacking the N-terminal 22 amino acids 16. It was reported that the Vtn receptor integrin on macrophages plays an essential role in phagocytosis of apoptotic neutrophils and inhibits the recognition of apoptotic cells by macrophages 17. Subsequent studies have also shown that Vtn can independently affect the macrophages and the apoptotic cells to reduce the clearance of apoptotic cells 18. Nonetheless, the mechanism and the potential importance of the Vtn-C1qbp ligand-receptor pair for phagocytosis remain unknown.

In subsequent experiments, we explored the effects and the underneath mechanism of Vtn-C1qbp regulating macrophage phagocytosis as well as tumor growth. Altogether, our work reveals a new regulatory ligand-receptor pair between macrophages and tumor cells, and their playing role in tumor growth as well as tumor microenvironment remodeling, providing new therapeutic targets for cancer immunotherapy.

## Methods

### Cell culture

Raw264.7 and HEK293T were purchased from the American Type Culture Collection (ATCC, Washington, USA). Mouse breast cancer cell line 4T1 was obtained from Dr R.A. Reisfeld (The Scripps Research Institute, La Jolla, CA, USA). Raw264.7 and HEK 293T cells were cultured in Dulbecco's modified Eagle's medium (DMEM) with high glucose (Biological Industries). 4T1 cells were cultured in RPMI 1640 (Biological Industries). All the mediums were supplemented with 10% fetal bovine serum (Biological Industries), 100 U/mL penicillin, and 100 mg/mL streptomycin (Gibco, Grand Island, NY). Cells were maintained at an atmosphere of 5% CO_2_ and 95% air at 37 ˚C.

Bone marrow cells (BMCs) were isolated from the femur and tibia of 6-week-old BALB/c mice. The BMCs were cultured in RPMI 1640 supplemented with 30 ng/mL recombinant mouse M-CSF (96-315-02-100, Peprotech). The medium was changed every 3 days. BMCs were differentiated into BMDMs by stimulation with M-CSF for 7 days.

### Establishment of stable cell lines

Vtn and C1qbp shRNAs were constructed by inserting the shRNA templates into the lentivirus-based RNAi vector PLKO.1 (Addgene, MA). The sequences for shRNAs targeting mouse Vtn and C1qbp are: shVtn1, F-CCGGGGTTTCTCTGGCTGACCAAGACTCGAGTCTTGGTCAGCCAGAGAAACCTTTTTG, R-AATTCAAAAAGGTTTCTCTGGCTGACCAAGACTCGAGTCTTGGTCAGCCAGAGAAACC; shVtn2, F-CCGGGCAGTACTGCTATGAGCTAGACTCGAGTCTAGCTCATAGCAGTACTGCTTTTTG, R-AATTCAAAAAGCAGTACTGCTATGAGCTAGACTCGAGTCTAGCTCATAGCAGTACTGC; shC1qbp1, F-CCGGGGCCTTCGTTGAATTCTTGACCTCGAGGTCAAGAATTCAACGAAGGCCTTTTTG, R-AATTCAAAAAGGCCTTCGTTGAATTCTTGACCTCGAGGTCAAGAATTCAACGAAGGCC; shC1qbp2, F-CCGGTGAGATTGGACACGAAGATGACTCGAGTCATCTTCGTGTCCAATCTCATTTTTG, R-AATTCAAAAATGAGATTGGACACGAAGATGACTCGAGTCATCTTCGTGTCCAATCTCA. The coding sequence of mouse Vtn and C1qbp was cloned into plasmid plenti-CMV-MCS-puro (Addgene) to generate their cDNA expression vectors. All plasmids were validated by sequencing. The lentiviral plasmids and packaging plasmids were co-transfected into 293T cells by Lipofectamine™ 2000 Transfection Reagent (ThermoFisher Scientific, USA), and lentiviruses produced by 293T cells were collected. Cells infected with lentiviruses for RNAi or cDNA expression were selected with 4 mg/mL of puromycin (ThermoFisher Scientific, USA).

### Flow cytometry-based phagocytosis assay

All in vitro phagocytosis assays reported here were performed by co-culturing tumor cells and macrophages at a ratio of 200,000 tumor cells to 100,000 macrophages for 2 h in a humidified, 5% CO_2_ incubator at 37 °C in ultra-low-attachment 96-well U-bottom plates (Corning). Tumor cells expressing fluorescence GFP were collected from plates using TrypLE Express (Life Technologies) before co-culture. After co-culture, phagocytosis assays were stopped. Cells were centrifuged at 400 g for 5 min at 4 °C and stained with APC-labelled anti-CD45 (Clone I3/2.3, BioLegend) to identify macrophages. Assays were analyzed by flow cytometry on Guava easyCyte (Millipore). Phagocytosis was measured as the number of CD45^+^GFP^+^ macrophages, quantified as the percentage of the total CD45^+^macrophages.

### In vivo phagocytosis analysis

For 4T1 phagocytosis analysis, 5 × 10^5^ of 4T1^shNC^-GFP^+^ cells or 4T1^shVtn1/2^-GFP^+^ cells were engrafted into 6-8-week-old female BALB/c mice via injection orthotopically into the mammary fat pad. Tumors were allowed to grow for 18 days, after which tumors were resected and dissociated mechanically and enzymatically. Single-cell suspensions of tumors were stained for flow cytometry analysis. Phagocytosis was measured as the percentage of CD11b^+^F4/80^+^ TAMs that were also GFP^+^.

### CRISPR/Cas9 knockout screening

The pooled lentiviral mouse CRISPR Brie libraries were purchased from Addgene (Watertown, MA) and amplified as described 19. The library contains 78637 sgRNAs targeting 19,674 genes and 1000 non-targeting sgRNAs. Lentiviruses carrying the library were generated and used to infect Raw264.7 or 4T1-GFP with the multiplicity of infection (MOI) of around 0.5. After selection with puromycin, 2 million Raw264.7 cells or 4T1-GFP cells expressing sgRNAs were subjected to phagocytosis assays as described in flow-cytometry-based phagocytosis assay. Phagocytosis-positive (CD45^+^GFP^+^) populations were sorted using Sony MA900. Genomic DNA was extracted using the DNAeasy kit (QIAGEN), and the cassettes containing the sgRNA sequence were amplified by one round of PCR following the procedure described 19. The amplified samples were sequenced on a HiSeq2000 (Illumina). The MAGeCK was used to rank genes based on redundant targeting guides via robust rank aggregation (RRA) 20. The RRA score is from 0 to 1, with closer to 0 indicating a stronger correlation.

### Western blotting analysis

Cells were lysed in radioimmunoprecipitation assay (RIPA) lysis buffer with protease inhibitor cocktail (Roche Life Science, Switzerland) and phosphatase inhibitor cocktail (Sigma-Aldrich). The concentrations of proteins were measured by Pierce BCA Protein Assay Kit (Thermo Fisher Scientific). 20 μg of total protein were loaded and separated on Tris-acrylamide gels. Proteins were transferred to polyvinylidene fluoride (PVDF) membranes (Merck). The membranes were blocked with 5% defatted milk before blotted with primary antibodies and secondary antibodies (ZSGB-BIO, China). The membranes were incubated with Immobilon Western Chemiluminescent HRP Substrate (Merck), and visualized and photographed by Tanon-5200 (Tanon, Shanghai, China).

### Murine models

All the animals were handled according to approved Institutional Animal Care and Use Committee protocols of Nankai University. Animal experiments were approved by Institutional Animal Care and Use Committee of Nankai University (ethic approved number: 20180014). All attempts were made to minimize the handling time during surgery and treatment so as not to unduly stress the animals. Animals were observed daily after surgery to ensure there were no unexpected complications. 6-8-week-old female BALB/c mice were purchased from Charles River (Beijing, China). For in vivo test of tumor inhibition with Vtn-knockdown, 5 × 10^5^ of 4T1^shNC^ cells or 4T1^shVtn1/2^ cells suspended in 100 μL phosphate buffered saline (PBS) were orthotopically injected into the fourth mammary fat pad. The tumor size was measured and recorded on the 7th day after injection. On day 28 after injection, tumor, lung, and spleen in each mouse were harvested and tested by flow cytometry, hematoxylin, and eosin (H&E) staining, and immunohistochemical (IHC) analyses. For in vivo tests of tumor inhibition with the CD47 antibody, 5 × 10^5^ of 4T1^shNC^ cells or 4T1^shVtn2^ cells suspended in 100 μL phosphate buffered saline (PBS) were orthotopically injected into the fourth mammary fat pad. The tumor size was measured and recorded on the 7th day after injection. 100 μL PBS containing 150 μg of control IgG or the anti-CD47mAb were intraperitoneal injected from day 7 and repeated every other day until the end of experiments. On day 26 after injection, tumor, lung, and spleen in each mouse were harvested and tested by flow cytometry, H&E staining, and IHC analyses.

### Flow cytometry

For 4T1 tumor-bearing mice, tumors were harvested, and cells were recovered by digesting the tumors with collagenase/DNase for 60 min at 37 °C, before crushing them on 70 µm cell strainers. All cells were then stained in FACS buffer, supplemented with 4% normal mouse sera, using a combination of fluorescent monoclonal antibodies. Suspensions of single cells were incubated with the antibodies for 30 min in the dark and washed twice using PBS with 1% FBS. Guava easyCyte (Millipore) was used to obtain the data, and FlowJo software (BD Biosciences) was used to analyze the data. Antibody information is described in Supplementary tables S1.

### qRT-PCR analysis

Total RNA was extracted using Trizol reagent (ThermoFisher Scientific, USA). 1 μg of total RNAs from cells or tissues were reverse transcribed into cDNAs using All-in-One First-Strand cDNA Synthesis SuperMix for qPCR regent (Transgen Biotech). Quantitative PCR analysis was performed using SYBR Green SuperMix (Transgen Biotech) on CFX96 system (Biorad) with the program as follows: 95 ˚C for 300 s, 45 cycles of two-step reaction, that is, 95 ˚C for 30 s followed by 60 ˚C for 45 s. The primer sequences are listed in Supplementary tables S2. The relative gene expression was calculated by the 2^-∆∆Ct^ method using GAPDH for normalization.

### H&E staining, IHC, and IF

Tissues dissected from mice were fixed in 4% paraformaldehyde, dehydrated, paraffin-embedded, serially sectioned to a thickness of 7 mm, and then de-paraffinized. For H&E staining, tissue sections were stained with hematoxylin and eosin. For IHC staining, after treatment with antigen retrieval solution and 3% hydrogen peroxide, tissue sections were blocked with 5% goat serum and incubated with primary antibody, biotin-conjugated secondary antibody, and streptavidin. Sections were visualized with 3,30-diaminobenzidine (DAB) substrate (ZSGB-BIO) followed by counterstaining with hematoxylin. Images were visualized using an Olympus microscope (Tokyo, Japan). For IF staining, tissue sections were blocked with 5% goat serum, incubated with primary antibody, secondary antibody conjugated with Alexa Fluor-488 or Alexa Fluor-647 (Thermo Fisher Scientific), and then counterstained with 40,6-diamidino-2-phenylindole (DAPI, Sigma-Aldrich). Images were visualized using TCS-SP8 STED 3X (Germany).

### CM stimulation and co-culture system

To investigate the role of Vtn in regulating the polarization of macrophages, 4T1^shNC^, 4T1^shVtn1^, 4T1^shVtn2^, or 4T1^VtnOE^ cells were directly co-cultured with Raw264.7 cells at a ratio of 2:1 for 48 h. To investigate the role of C1qbp in regulating the polarization of macrophages, Raw264.7^shNC^ or Raw264.7^shC1qbp^ cells were directly co-cultured with 4T1 cells at a ratio of 1:2 for 48 h. The macrophages were then collected in TRIzol reagent for RNA extraction.

### Co-immunoprecipitation

Cell pellets were lysed with coimmunoprecipitation buffer (Solarbio) supplemented with protease inhibitors. Insoluble material was removed by centrifugation for 10 min at 13,000 g at 4 °C. Protein A/G Magnetic Beads (50 μL) (MedChemExpress) were preincubated overnight with 10 μg of the indicated antibodies. Antibody-coated beads were washed with binding/wash buffer (1× PBS + 0.5% Tween-20, pH 7.4), and incubated with 5 mg of cleared lysate overnight at 4 °C. The beads were washed 4 times with binding/wash buffer. The immunoprecipitated proteins were eluted in 50 μL of elution buffer (0.15 M Glycine, pH 2.5-3.1) and boiled for 5 min. The final samples were separated by SDS-PAGE and transferred onto PVDF membrane for immunoblotting.

### Mass spectrometry (MS) analysis

Whole-cell lysates were prepared from Raw264.7 cells. The cell lysates were preincubated with anti-C1qbp antibody or control IgG overnight at 4 °C. And the mixtures were incubated with Protein A/G Magnetic beads for 4 h. The Interaction complexes were analyzed using LC/MS/MS on Orbitrap Fusion Lumos Tribrid. Production data were searched against the NCBI protein database using the Mascot and X-Tandem search engines. Mascot output files were parsed into the Scaffold program (www.proteomesoftware.com) for filtering to assess false discovery rates and allow only correct protein identifications.

### Bioinformatics analysis

From the Genomic Data Commons (GDC) of TCGA, the clinical information and sequencing data of breast cancer patients were downloaded at the UCSC Xena browser (https://xenabrowser.net) and sorted into a matrix. Patients were divided into two groups according to the expression of Vtn. The group with the highest quartile of expression levels was labeled as the high-expression group, while the remaining patients were assigned to the low-expression group. The Kaplan-Meier method was used to generate survival curves. The log-rank test was used to assess statistical significance between different groups. Differences between groups were analyzed using an unpaired two-tailed t-test. The TCGA and transcriptomic data were processed through CIBERSORT (https://cibersort.stanford.edu/) using the default LM22 immune cell gene signatures.

For scRNA sequencing data processing, scRNA-seq data of 9 TNBC patients were downloaded from GSE176078 on the Gene Expression Omnibus (GEO), and 35531 cells were selected. The cell types in scRNA-seq were used as indicated in the original article 21. Macrophages were classified based on their C1QBP expression level whereby a C1QBP^+^ macrophage refers to a macrophage with detectable expression of C1QBP and a C1QBP^-^ macrophage indicates no detectable expression. For the cell-to-cell interaction analysis, ligand and receptor interactions were analyzed using CellPhoneDB (https://www.cellphonedb.org) package. Dot plots depicting the interactions between macrophage and cancer cells were drawn using ktplots package in R.

### Statistics

Prism 8.0 software (GraphPad Software, San Diego, CA, USA) was used for statistical analysis. Statistical parameters including the definitions and exact values of n, statistical test, and statistical significance are provided in the figures and figure legends. Quantitative data were presented as means ± SEM, and the differences between the groups were analyzed using the student's t-test. Survival curves were analyzed using the Kaplan-Meier method, and the log-rank test was used to calculate the differences between the curves. Differences are considered statistically significant at *p < 0.05; **p < 0.01; ***p < 0.001; ns means no significance, p > 0.05.

### Data availability

The data underlying Figure 2, Figure 3, and Figure S2, Figure S3 are openly available in TCGA-BRCA patient's data at the UCSC Xena browser (https://xenabrowser.net). The data underlying Figure S9 is openly available in GEO at GSE176078. The remaining data generated or analyzed during the current study are available within the article and its Supplementary Data files.

## Results

### A genome-wide CRISPR screen in macrophages and tumor cells identifies regulators of phagocytosis

**Figure 1:**
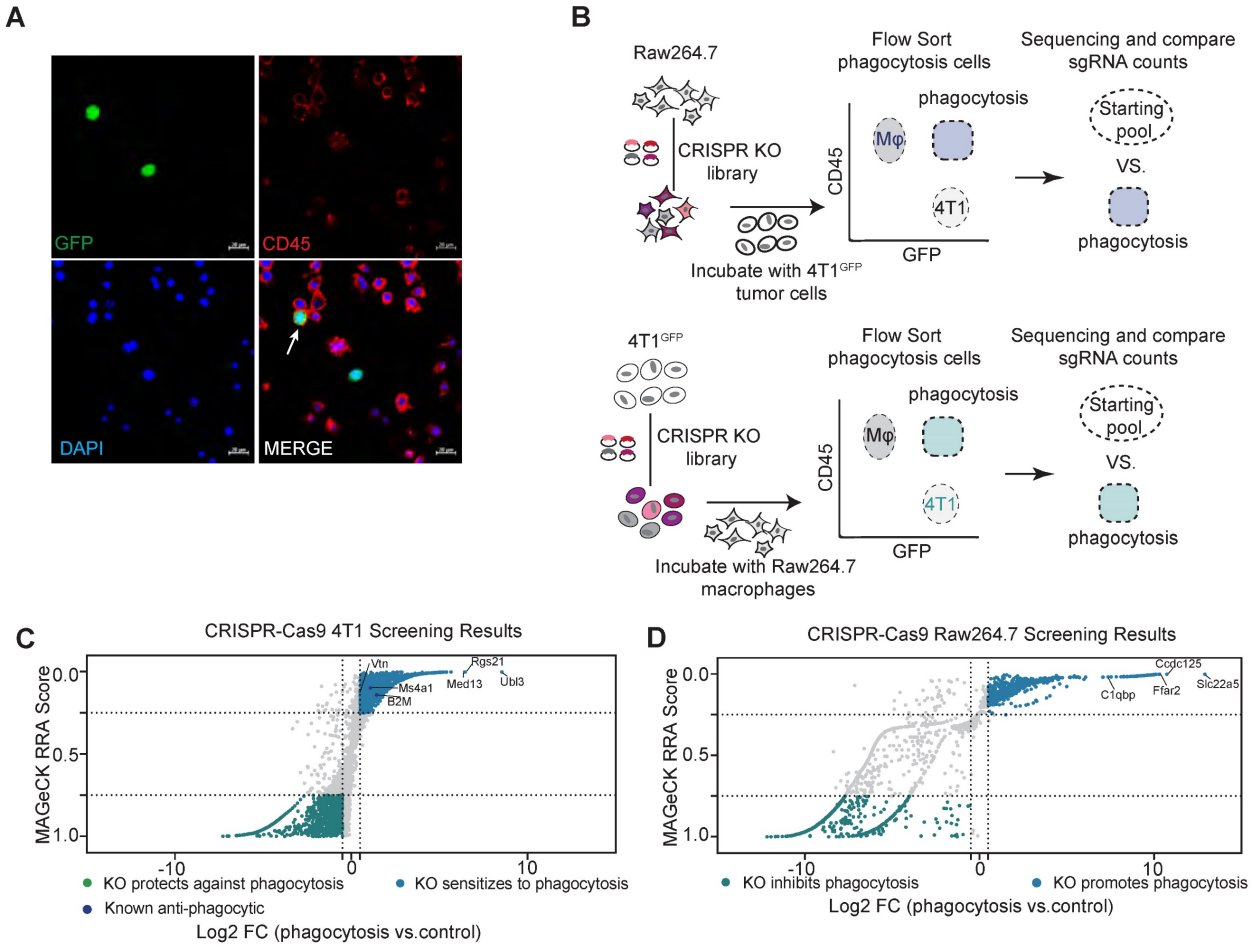
** Genome-wide CRISPR screening for regulators of phagocytosis. (A)** Representative phagocytosis images of Raw264.7 macrophages after 2 h incubation with GFP-labelled-4T1 cells. The white arrow indicates phagocytosis. Scale bars, 20 µm. **(B)** Schematic representation of the major steps of the genome-wide screen to identify inhibitory phagocytosis-related genes in 4T1 cells and Raw264.7 cells. **(C and D)** A scatterplot of the 4T1 (C) and Raw264.7 (D) screen results compared to the untreated control, showing positive regulators and negative regulators. Genes that met the cutoff criteria (MAGeCK RRA score < 0.25 and Log2 FC > 0.5) are shown as blue dots for positive regulators and met the cutoff criteria (MAGeCK RRA score > 0.75 and Log2 FC < -0.5) as green dots for negative regulators.

To systematically search for ligand-receptor interactions that regulate macrophage phagocytosis of tumor cells, we transduced macrophages and tumor cells separately with a mouse genome-wide lentiviral CRISPR sgRNA library (lentiCRISPRv2-Brie) for a loss-of-function screen 19. We selected a mouse macrophage cell line, Raw264.7, which has similar characteristics to that of normal macrophages, with phagocytic activity and anti-tumor activity 22. In addition, the effectiveness of CD47-targeted therapy in treating solid tumors in clinical trials, particularly in the case of triple-negative breast cancer (TNBC), has been found to be less than satisfactory, suggesting the TNBC cells can escape from phagocytic elimination by macrophages through CD47-independent mechanisms 23, 24. Therefore, we chose 4T1 cells as the tumor cell model for our screen. To visualize the phagocytosis of macrophages, we transduced 4T1 cells with GFP (Green fluorescent protein) so that macrophage phagocytosis of tumor cells can be detected by Fluorescence Activated Cell Sorter (FACS) (Figure 1A). We have thus established a FACS-based phagocytosis assay for rapidly sorting out Raw264.7 cells with the ability to phagocytose tumor cells, to be used for identification of genes regulating macrophage phagocytosis of tumor cells.

Using this assay, we performed a genome-wide screen for phagocytosis-regulating genes (Figure 1B). Raw264.7 was transduced with retroviral sgRNA library at a multiplicity of infection (MOI) of 0.5 to ensure that only one sgRNA was transduced per cell. sgRNA-transfected Raw264.7 cells were co-cultured with 4T1 cells for phagocytosis assays in vitro. After two hours of co-culture, CD45^+^GFP^+^ phagocytosis cells were sorted and collected. sgRNA sequences within the genomic DNA of sorted cells were recovered by PCR amplification and identified by high-throughput sequencing. The relative enrichment of sgRNAs in phagocytotic vs. all macrophages was calculated by the MAGeCK (model-based analysis of genome-wide CRISPR-Cas9 knockout) algorithm 20. Similarly, 4T1 cells were subjected to the same screening process to identify genes regulating macrophage phagocytosis in cancer cells.

sgRNAs enriched in phagocytotic cell populations (denoted by RRA score closer to 0) are those causing increased phagocytosis of tumor cells by macrophages when disrupted, and therefore represent genes that normally inhibit phagocytosis. By using the criteria of log2(fold change) > 0.5 and RRA score < 0.25, we identified 660 potential phagocytosis inhibitory genes enriched in macrophages and 1138 potential genes that mediate resistance to phagocytosis enriched in tumor cells (Figure 1C-D). As expected, we identified the known breast cancer anti-phagocytosis gene B2M (Beta-2-Microglobulin) and the well-known antibody therapeutic target MS4A1 (CD20) in tumor cells 4, 25. KEGG pathway enrichment analysis of the phagocytotic resistance genes identified from tumor cells were mainly enriched in metabolic pathways, cell junctions, and major tumor signaling pathways such as Wnt, mTOR, AMPK, and PI3K-AKT signaling pathways (Figure S1A). In parallel, the phagocytosis inhibitory genes identified in macrophages showed strong enrichment of pathways related to phagocytosis such as metabolic, regulation of actin cytoskeleton, endocytosis, and lysosomal pathways (Figure S1B).

### Identification of the Vtn-C1qbp ligand-receptor pair as a regulator of macrophage phagocytosis of tumor cells

**Figure 2:**
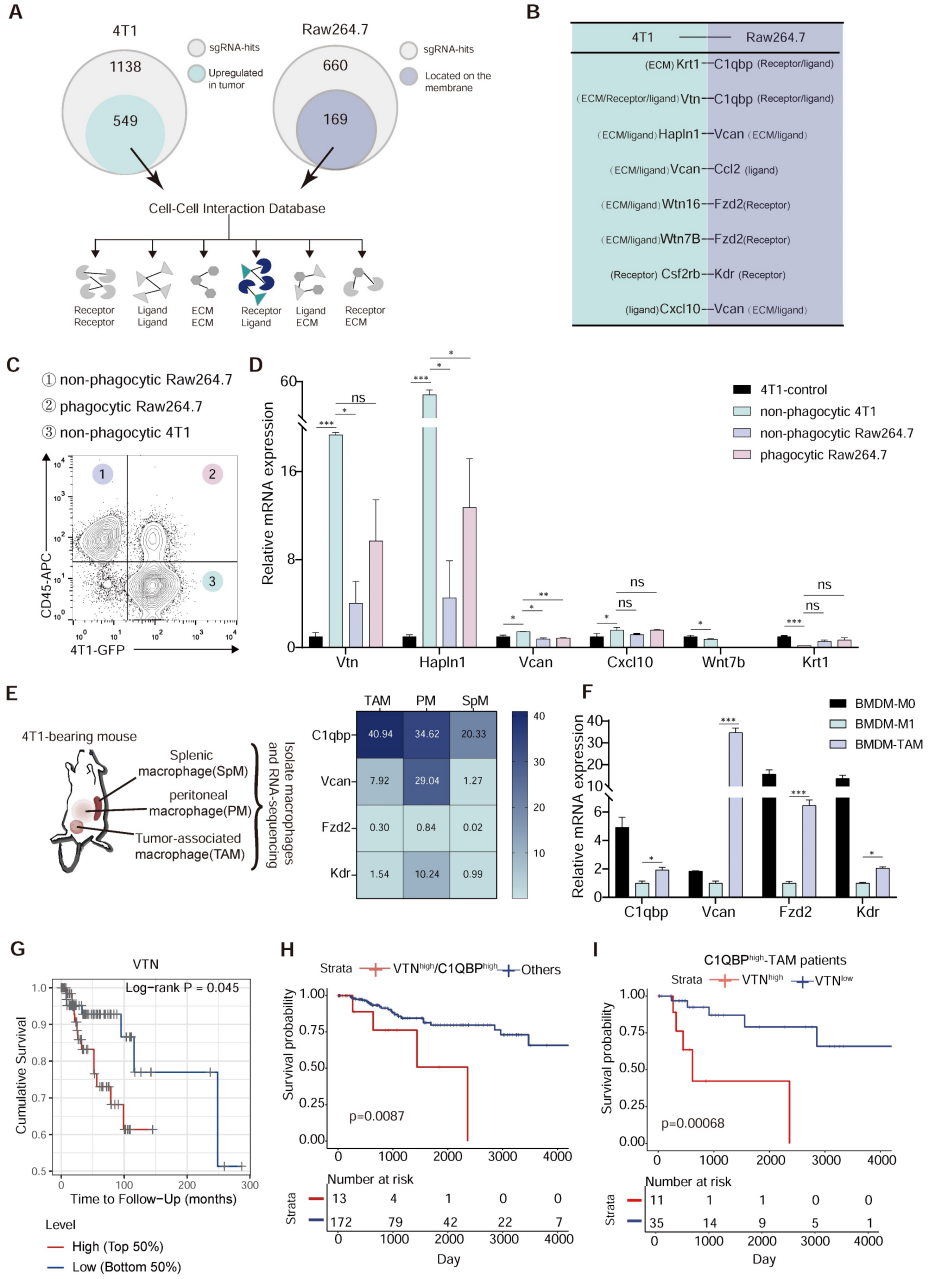
** Vtn-C1qbp ligand-receptor pair as a putative modifier of phagocytosis. (A)** Diagram of criterion for screening candidate cell-cell interactions. First, the tumor-derived candidate genes that upregulated in TCGA-BRCA tumor tissues compared to normal tissues were selected from positive sgRNA hits in 4T1 and macrophage-derived candidate genes which could be located on the membrane were selected from positive sgRNA hits in Raw264.7. Next, mapping these two parts of the gene to the cell-cell interaction database to screen out candidate interactions between Raw264.7 and 4T1. **(B)** Selected candidate interactions filtered according to the screen criteria. **(C)** Diagram of FACS-based phagocytosis. GFP^+^CD45^-^ 4T1 cells that have not been phagocytosed: non-phagocytic 4T1; GFP^+^CD45^+^: phagocytic Raw264.7; GFP^-^CD45^+^: non-phagocytic Raw264.7. **(D)** qRT-PCR analysis of 4T1 candidate genes as shown in (B) in non-phagocytic 4T1, phagocytic Raw264.7, non-phagocytic Raw264.7 and control 4T1 cells. **(E)** Heatmap (based on FPKM values) of the Raw264.7 candidate genes in (B) on SpM, PM, and TAM from 4T1 tumor-bearing mouse. Color intensity denotes level of gene expression. SpM: Splenic macrophage; PM: Peritoneal macrophage; TAM: Tumor-associated macrophage. **(F)** qRT-PCR analysis of Raw264.7 candidate genes as shown in (C) in BMDM-M0 (unstimulated), BMDM-M1 (stimulated with LPS), and BMDM-TAM (stimulated with conditioned medium of 4T1 cells). BMDM: bone marrow-derived macrophage. **(G)** Overall survival of TCGA patients with basal breast cancer (n = 140) with high or low VTN expression as defined by the median. Two-sided P value computed by a log-rank (Mantel-Cox) test. **(H)** Survival curves comparing combined high expression of VTN and C1QBP with other patients. Two-sided P value computed by a log-rank (Mantel-Cox) test. Numbers of subjects at risk in the high group (red) compared with the other group (blue) are indicated below the x-axes. **(I)** Survival curves comparing high or low expression of VTN in C1QBP^high^ macrophage infiltration patients (n = 46) in TCGA-TNBC database. Two-sided P value computed by a log-rank (Mantel-Cox) test. Numbers of subjects at risk in the high group (red) compared with the other group (blue) are indicated below the x-axes. All the qRT-PCR data are shown as means ± SEM from three independent experiments, *p < 0.05, **p < 0.01, ***p < 0.001, ns not significant, by unpaired two-sided Student's t-test.

To find the inhibitory ligand-receptor pairs that exist in macrophages and tumor cells, we selected genes that were identified as inhibitors of phagocytosis in the CRISPR screen. Among the 660 hits identified from Raw264.7 cells, 169 encoded cell surface proteins which are candidates for the receptor. We also reasoned that the anti-phagocytosis ligands mediating evasion from macrophage clearance are likely overexpressed in tumor cells. Thus, among the 1138 hits from 4T1 cells, we focused on the 589 genes with increased expression in breast cancer tissues as compared to normal tissues in breast cancer patients in the TCGA database (Figure 2A and Figure S2A), as candidates for the ligand. We then mapped these candidate genes from Raw264.7 and 4T1 cells to the cell-cell interaction database from the BADER lab in the University of Toronto, which contained 1851 receptors, 1593 ligands and 433 extracellular matrix components, representing 38446 cell-cell interactions, and identified 8 intercellular interactions among our candidates (Figure 2B). To further narrow down the candidate interactions, we sorted out the non-phagocytic 4T1 cells, phagocytic Raw264.7 cells, and non-phagocytic Raw264.7 cells after 4T1 co-culture with Raw264.7 for 2 h by flow cytometry (Figure 2C). By detecting the expression levels of Vtn, Hapln1, Vcan, Cxcl10, Wnt7b and Krt1, we found that the expression of Vtn and Hapln1 was significantly elevated in non-phagocytic 4T1 as compared to 4T1 cells not co-cultured with Raw264.7, while the levels of Vcan, Cxcl10, Wnt7b and Krt1 were not significantly changed (Figure 2D). In addition, we performed RNA sequencing of tumor-associated macrophages (TAMs), splenic macrophage (SpM) and peritoneal macrophage (PM) in 4T1 tumor-bearing mice, and found that Fzd2 (Frizzled Class Receptor 2) and Kdr (Kinase Insert Domain Receptor) were barely expressed in macrophages, and that C1qbp was highly expressed in tumor-associated macrophages, while Vcan was not highly expressed in TAMs (Figure 2E). Subsequently, we induced bone marrow-derived macrophages (BMDMs) into M1-like (using LPS) and TAM (using 4T1 cell supernatant) in vitro, and detected the expression of these genes. Based on the CT values of genes, we observed weak expression of Fzd2 and Kdr in BMDMs, while C1qbp and Vcan exhibited high expression. Meanwhile, C1qbp and Vcan were highly expressed in TAMs but not in M1-like BMDMs (Figure 2F and Figure S2B). Therefore, C1qbp and Vcan were selected as candidate receptor genes on macrophages.

As our studies revealed two candidate ligand-receptor pairs, Vtn-C1qbp and Hapln1-Vcan, we further analyzed the correlation between the levels of Vtn and Hapln1 and the prognosis of basal-like breast cancer. Stratification of patients by gene expression revealed an overall survival advantage for breast cancer patients with lower Vtn expression or higher Hapln1 expression (Figure 2G and Figure S2C). By analyzing TCGA bulk tissue RNA profiles, we found that dual enhancement of VTN and C1QBP was associated with a worse prognosis in basal-like breast cancer patients compared to patients with high or low expression of either gene (Figure 2H).

We also found that infiltration of C1QBP^high^ macrophages was significantly associated with poor prognosis of TNBC patients. Further analysis showed that among patients with C1QBP^high^ macrophage infiltration, the combination together with high expression of VTN was more significantly associated with poor prognosis than in patients with C1QBP^low^ macrophage infiltration (Figure 2I). These results reinforce a synergistic negative effect of VTN and C1QBP-expressing macrophages in patients with TNBC (Figure S3A-B). Therefore, based on these results, the ligand-receptor pair Vtn-C1qbp was selected for further investigation.

### C1qbp is the main binding molecule of Vtn on the macrophage membrane

**Figure 3:**
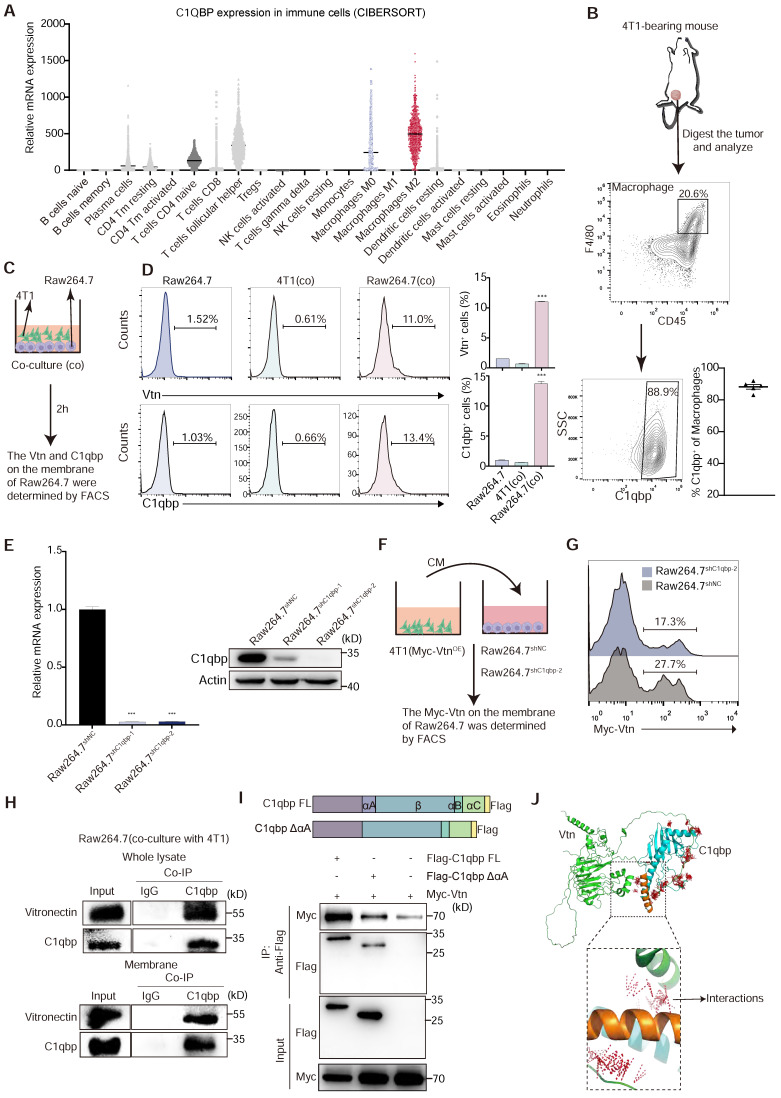
** Vtn binds to C1qbp on the plasma membrane of macrophages. (A)** Relative C1QBP mRNA levels in immune cell subsets assessed by CIBERSORT in patients with breast cancer from TCGA. **(B)** Gating strategy for C1qbp^+^ TAMs in 4T1 tumors; C1qbp^+^ TAMs were assessed as CD45^+^F4/80^+^C1qbp^+^, right, frequency of C1qbp^+^ TAMs in 4T1 tumor-bearing mice (n = 5).** (C)** Schematic of the in vitro co-culture experiment to detect the C1qbp and Vtn on the surface of 4T1 and Raw264.7 cells. **(D)** Flow cytometry analysis of Vtn and C1qbp expression on the surface of 4T1 and Raw264.7 cells after co-culture. The quantification is shown in (D) as means ± SEM (n = 3), ***p < 0.001, by unpaired two-sided Student's t-test. **(E)** qRT-PCR and Western blotting analyses showing the knockdown efficiencies of C1qbp shRNA in Raw264.7 cells. **(F)** Schematic of the in vitro crosstalk experiment using conditioned medium (CM) from 4T1 (Myc-Vtn^OE^) cells to stimulate macrophages. **(G)** Flow cytometry analysis of Myc-Vtn on the surface of C1qbp-knockdown Raw264.7 cells after stimulated with Myc-Vtn contained CM. Numbers indicate the frequency of Myc-Vtn^+^ events out of total cells. **(H)** Co-immunoprecipitation of Vtn and C1qbp in whole lysate and membrane of Raw264.7 stimulated with conditioned medium (CM) of 4T1 cells. **(I)** Schematic diagram of C1qbp deletion mutants and their genetic complementation phenotypes. The full-length C1qbp (C1qbp FL) and C1qbpΔαA (lacks the αA domain) are tagged with Flag. Defining the Vtn-interacting domain in C1qbp. Co-IP and western blotting of HEK293T cells co-transfected with Myc-tagged Vtn and a vector expressing the indicated Flag-tagged C1qbp deletion mutants or full-length C1qbp. **(J)** The molecular docking complexes of C1qbp domain (cyan, αA domain was orange) and full-length Vtn protein (green) as obtained from ClusPro. The amino acids binding interaction show as red line.

To assess the role of Vtn-C1qbp signaling in regulating the macrophage-mediated immune response to cancer, we examined the expression of C1qbp in immune cells. Analysis using two deconvolution algorithms, Tumor Immune Estimation Resource (TIMER) and CIBERSORT 26, 27, with RNA-sequencing data from The Cancer Genome Atlas (TCGA), revealed high expression of C1QBP in macrophage, particularly in immunosuppressive M2-like macrophages among various cell types in the human breast cancer microenvironment (Figure 3A). Additionally, our FACS analysis showed that the membrane-bound form of C1qbp was primarily present in macrophages, rather than tumor cells, within the microenvironment of 4T1 tumors (Figure S3C-D). In addition, TAMs infiltrated into 4T1 tumors are almost all membrane-C1qbp^+^, suggesting that membrane-C1qbp^+^ macrophages may play an immunosuppressive function (Figure 3B).

Vtn is typically a secreted protein 13, while C1qbp can be localized on the plasma membrane or intracellularly 28. To investigate the effect of Vtn-C1qbp signaling on phagocytosis, we detect the presence of Vtn and C1qbp on macrophage membranes during phagocytosis (Figure 3C).

FACS results show that direct co-culture of Raw264.7 and 4T1 cells for 2 h significantly augments the proportion of C1qbp^+^ and Vtn^+^ macrophages, as compared to 4T1 cells and Raw264.7 cells not co-cultured with 4T1 (Figure 3D). To further confirm the binding of tumor-derived Vtn with C1qbp on macrophage plasma membranes, we co-cultured the control Raw264.7 or C1qbp-knockdown Raw264.7 cells, in which C1qbp was hardly detectable (Figure 3E), with the conditional medium (CM) from 4T1 cells expressing Myc-tagged Vtn (Figure 3F). As expected, in the absence of C1qbp, Myc-Vtn binding to the plasma membrane of Raw264.7 was significantly diminished, indicating that C1qbp is a major receptor for Vtn on the plasma membrane of macrophages (Figure 3G). To further validate the binding of Vtn to C1qbp, we performed immunoprecipitation (IP) coupled with Western blotting analysis in Raw264.7 cells. Vtn co-immunoprecipitated with C1qbp in the whole cell lysates as well as the membrane fractions of Raw264.7 cells co-cultured with 4T1 (Figure 3H). It has been reported that the binding of the multimeric Vtn to C1qbp is significantly reduced after truncation of the N-terminal 22 amino acids of the mature form of C1qbp 16. To define the binding domain of Vtn to C1qbp, we constructed a mutant mouse C1qbp by truncating the first 20 amino acids (αA domain, nucleotides 229-288) at the N terminus of the mature form of C1qbp and adding Flag-tag at the C terminus. We performed co-IP assays in HEK293T cells co-transfected with Myc-Vtn and wild-type or mutant C1qbp. As shown in Figure 3I, Myc-Vtn specifically co-immunoprecipitated with the Full-length C1qbp, but to a substantially lesser extent with the mutant lacking the αA domain (amino acid residues 77-96), indicating that Vtn primarily interacts with the αA domain of C1qbp. The predicted crystallographic structures of C1qbp (AF-O35658-F1) and Vtn (AF-P29788-F1) were used for docking using the ClusPro server. The structure that C1qbp-αA domain dominant in binding with Vtn was selected and examined for the interaction between the two complexes (Figure 3J). These data suggest that the tumor-derived Vtn interacts with C1qbp mainly through the αA domain on the plasma membrane of macrophages.

### The Vtn-C1qbp axis protects cancer cells from phagocytosis by macrophages

**Figure 4:**
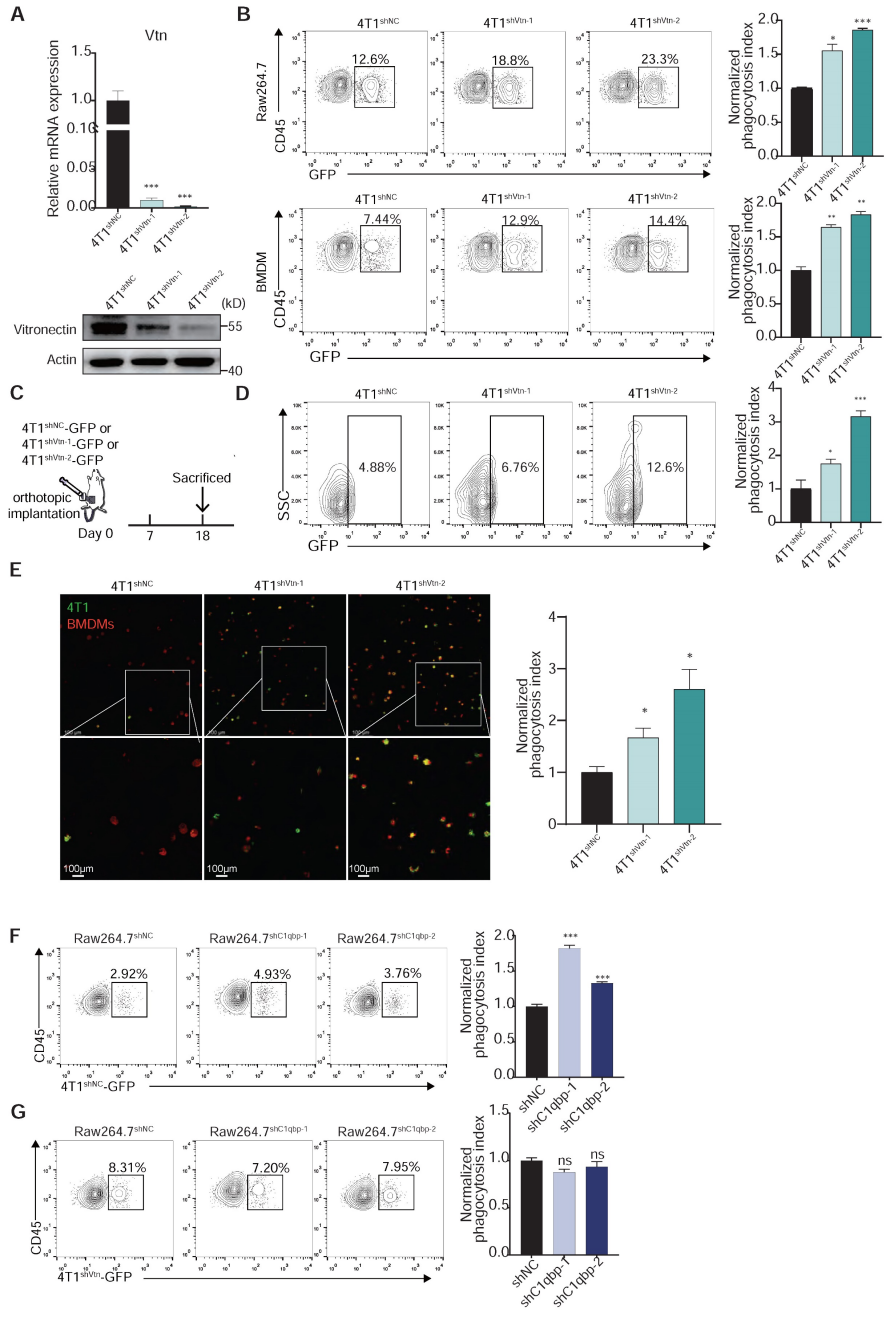
** Vtn-C1qbp inhibits phagocytosis of tumor cells by macrophage. (A)** qRT-PCR and Western blotting analyses showing the knockdown efficiencies of Vtn shRNAs. **(B)** Representative flow cytometry plots depicting the phagocytosis of control 4T1-GFP cells and Vtn-knockdown 4T1-GFP cells co-culture with Raw264.7 or BMDMs, numbers indicate frequency of phagocytosis events out of all macrophages. Data are plotted as means ± SEM from three independent measurements, *p < 0.05, **p < 0.01, ***p < 0.001, by unpaired two-sided Student's t-test. **(C)** Schematic of in vivo phagocytosis assay. GFP-labeled Vtn knockdown or control 4T1 cells were orthotopic injected into the mammary fat pad of BALB/c mice and sacrificed at day 18. **(D)** TAM phagocytosis is assessed as the frequency of CD11b^+^F4/80^+^GFP^+^ events out of total CD11b^+^F4/80^+^events. Numbers indicate frequency of phagocytosis events out of all macrophages. Plots are representative of three experimental replicates. *p < 0.05, **p < 0.01, ***p < 0.001, by unpaired two-sided Student's t-test. **(E)** Representative fluorescence microscopy images of in vitro phagocytosis of Vtn knockdown or control 4T1 cells (GFP^+^, green) by BMDMs (F4/80^+^; red) after 6 h of co-culture. Experiment was repeated with three times. Scale bar, 100 μm. *p < 0.05. **(F and G)** Representative flow cytometry plots demonstrating the phagocytosis of control 4T1-GFP cells (F) and Vtn-knockdown 4T1-GFP cells (G) by control or C1qbp-knockdown Raw264.7 cells, numbers indicate frequency of phagocytosis events out of all macrophages. Data are plotted as means ± SEM from three independent measurements, *p < 0.05, **p < 0.01, ***p < 0.001, ns not significant, by unpaired two-sided Student's t-test.

To investigate the role of Vtn-C1qbp signaling in regulating the macrophage-mediated anti-tumor immune response, we knocked down Vtn in 4T1 cells and conducted phagocytosis assay using Raw264.7 and BMDM (Figure 4A). Direct co-culture of either Raw264.7 or BMDM with control or Vtn-knockdown 4T1 cells revealed that Vtn knockdown alone was sufficient to potentiate phagocytosis (Figure 4B). To investigate whether the protection against phagocytosis conferred by Vtn could be recapitulated in vivo, GFP^+^ control or Vtn-knockdown 4T1 cells were orthotopically engrafted into mammary fat pad of BALB/c mice (Figure 4C). 18 days after engraftment, we found that Vtn-knockdown tumors exhibited augmented levels of in vivo phagocytosis by infiltrating TAMs as compared to control tumors (Figure 4D). To measure phagocytic clearance by confocal microscopy, control and Vtn-knockdown 4T1 cells were labelled with GFP and co-cultured with BMDMs for 6 h. Vtn-knockdown cells were more readily engulfed by macrophages as compared to the control cells (Figure 4E).

To investigate the effect of C1qbp expression on phagocytosis, we performed a phagocytosis assay using control and C1qbp-knockdown Raw264.7 cells. C1qbp-knockdown Raw264.7 cells demonstrated significantly greater phagocytic ability than control Raw264.7 (Figure 4F). We also found that silencing C1qbp had no effect on phagocytosis of Vtn-knockdown 4T1 cells (Figure 4G), suggesting that no other macrophage-secreted factors besides Vtn can stimulate the ability of C1qbp to suppress phagocytosis. Overall, these results suggest that Vtn-C1qbp is an anti-phagocytic signal that is capable of protecting tumor cells from phagocytosis by macrophages in vitro and in vivo.

### Vtn-C1qbp polarized macrophages toward an M2-like phenotype

To further investigate the effect of Vtn-C1qbp on tumor growth, we orthotopically inoculated control and Vtn-knockdown 4T1 cells in mice, and measured the tumor size every other day starting from day 7.

**Figure 5:**
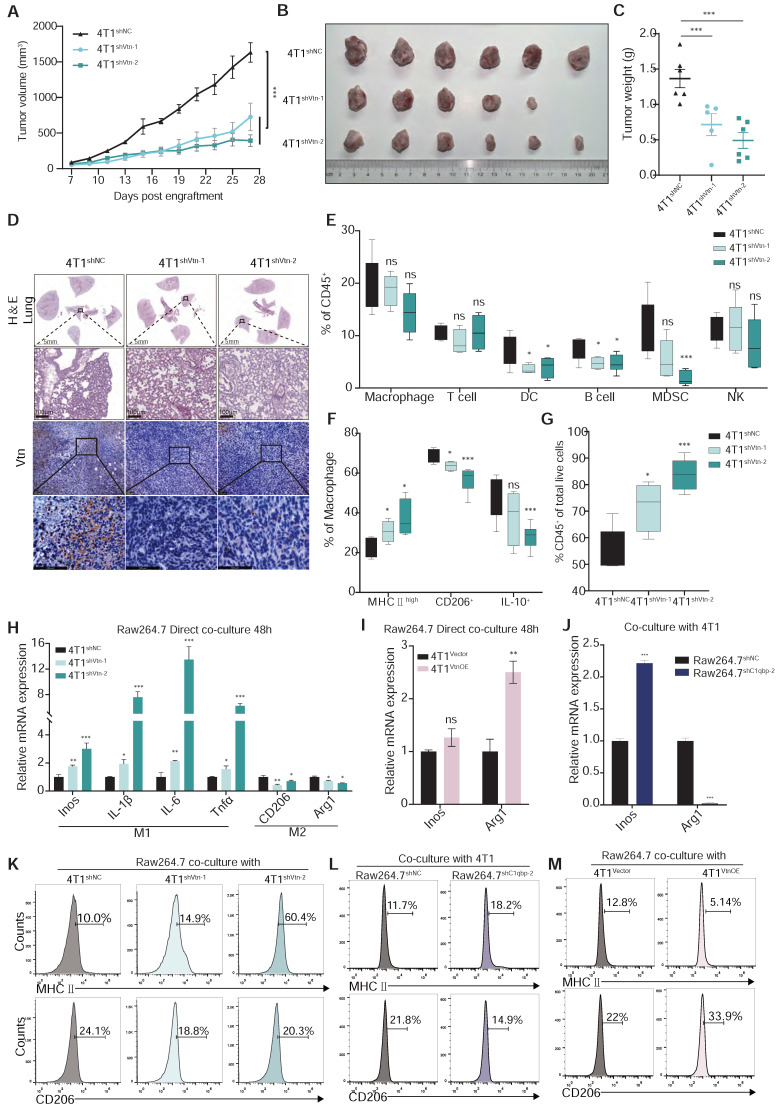
** Vtn-C1qbp differentiates macrophages towards an M2-like phenotype. (A)** The tumor growth curves after orthotopically inoculated with Vtn knockdown or control 4T1 cells. (n = 5-6, data are shown as means ± SEM, *p < 0.05, **p < 0.01 by unpaired two-sided Student's t-test). **(B)** Images of primary tumors dissected from BALB/c mice orthotopically transplanted with indicated 4T1 cells. **(C)** Weight of the collected tumors. (n = 5-6, data are shown as means ± SEM, *p < 0.05, **p < 0.01 by unpaired two-sided Student's t-test). **(D)** H&E-stained lung and tumor sections and immunohistochemistry of Vtn from animals orthotopically inoculated with indicated 4T1 cells. Scale bar = 100 μm. **(E to G)** Tumors from Vtn knockdown or control 4T1-bearing mice (n = 4-6) were harvested and analyzed by flow cytometry. Box plots show the percent of macrophages, T lymphocytes, dendritic cells, B lymphocytes, myeloid-derived suppressor cells (MDSCs), and NK cells in CD45^+^ cells (E), the percent of MHCⅡ^high^, CD206^+^ and IL-10^+^ in macrophages (CD11b^+^F4/80^+^) (F) and the percent of CD45^+^ cells (G) in total live events. The boxes extend from the 25th to 75th percentile, the horizontal line in the box represents the median, for the location of the whiskers the Min to Max method was chosen, *p < 0.05, ***p < 0.001, ns not significant, by unpaired two-sided Student's t-test. **(H)** qRT-PCR analysis of macrophage marker genes in Raw264.7 cells stimulated with Vtn knockdown or control 4T1 cells for 48 h. **(I and J)** qRT-PCR analysis of macrophage marker genes Inos and Arg1 in Raw 264.7 cells co-culture with Vtn-overexpression or control (Vector) 4T1 cells (I) and in control and C1qbp-knockdown Raw264.7 cells co-culture with 4T1 cells (J) for 48 h. **(K to M)** Flow cytometry analysis of M1-like (MHCII^+^) and M2-like (CD206^+^) subpopulations in Raw264.7 cells co-culture with Vtn knockdown or control 4T1 cells (K), with Vtn-overexpression or control (Vector) 4T1 cells (M) and in control and C1qbp knockdown Raw264.7 cells co-culture with 4T1 cells (L) for 48 h. All the qRT-PCR data are shown as means ± SEM from three independent experiments, *p < 0.05, **p < 0.01, ***p < 0.001, ns not significant, by unpaired two-sided Student's t-test.

We observed a robust reduction in the growth and weight of the Vtn-knockdown tumors as compared to the control tumors (Figure 5A-C). Splenomegaly (enlarged spleen), resulting from an increase in immature splenic granulocytes such as bone marrow-derived suppressor cells (MDSCs), was reported in 4T1 tumor-bearing mice 29. We also observed significant reduction in size and weight of spleen in Vtn-knockdown tumor-bearing mice compared to control mice (Figure S4A-B), and there was no notable difference in the body weight (Figure S4C). Lung is a major site of breast cancer metastasis. We found that Vtn knockdown in tumor cells led to a decrease in Vtn expression in the tumor tissues and a significant reduction in the number of metastatic sites in the lung (Figure 5D and Figure S4D). Vtn knockdown had no significant effect on the size and weight of the lungs (Figure S4E). We also examined the effect of Vtn-knockdown on its own proliferation and found that its proliferative capacity was unchanged compared to control (Figure S4F).

Macrophages are highly plastic and can polarize into M1-like pro-inflammatory or M2-like anti-inflammatory phenotypes. Tumor-associated macrophages that are conducive to tumor growth are of the M2-like type. To further investigate whether Vtn has an effect on the polarization of macrophages, we examined the phenotype of macrophages in the tumor microenvironment. In the syngeneic murine breast cancer model generated by orthotopic transplant of 4T1 cells, we analyzed the proportion of immune cells in the tumor, including macrophages, dendritic cells (DC), myeloid suppressor cells (MDSCs), and three major types of lymphocytes, T cells, B cells, and NK cells. There was no significant change in the proportion of macrophages (CD11b^+^F4/80^+^) between the control and Vtn knockdown mice (Figure 5E). However, compared to the control group, TAMs that infiltrated the Vtn-knockdown tumors has an increased proportion of M1-like macrophages (MHCⅡ^high+^), while the proportion of M2-like macrophages (CD206^+^ or IL-10^+^) decreased (Figure 5F). In addition, we observed a significant elevation in the proportion of leukocytes (CD45^+^) in Vtn-knockdown tumors as compared to control tumors (Figure 5G). The proportion of T lymphocytes and their subtypes CD4^+^ and CD8^+^, as well as NK cells, did not show significant changes in the control and Vtn knockdown tumors (Figure 5E and Figure S5A); However, CD4^+^ and CD8^+^ T cells infiltrated into the Vtn-knockdown tumors exhibited higher activation status (CD69^+^) as compared to control tumors (Figure S5B). The proportions of DC, B cells, and MDSCs were significantly decreased in Vtn-knockdown tumors (Figure 5E). Since the activation of CD4^+^ and CD8^+^ T cells are primarily dependent on antigen-presenting cells, and the decrease in the population of B cells and DC cells did not result in a reduction of activation, it is likely that increased TAM-mediated antigen presentation by phagocytosis of tumor cells was responsible for the enhanced activation of CD4^+^ or CD8^+^ T cells.

To further confirm the effect of Vtn-C1qbp axis on macrophage polarization, we co-cultured of Raw264.7 cells and 4T1 cells directly in vitro for 48 h and subsequently detected the macrophage phenotype. Macrophages co-cultured with Vtn-knockdown 4T1 cells expressed higher levels of M1-like markers and lower levels of M2-like markers than those co-cultured with the control 4T1 cells (Figure 5H).

We also found that macrophages were significantly biased toward M2-like after direct co-culture with the Vtn-overexpressing 4T1 cells as compared to those with control 4T1 cells (Figure 5I and Figure S6A). Meanwhile, C1qbp-knockdown Raw264.7 cells co-cultured with 4T1 cells expressed higher levels of M1-like markers and lower levels of M2-like markers as compared to the control Raw264.7 cells (Figure 5J). Similar results were obtained when the macrophage phenotypes were analyzed by flow cytometry. Silencing of Vtn or C1qbp increased the population of M1-like (MHCII^+^) macrophages and decreased that of M2-like (CD206^+^) macrophages (Figure 5K-L and Figure S5C-D), while overexpression of Vtn had the opposite effects (Figure 5D and Figure S5E).

To further validate the effect of Vtn-C1qbp signaling on the M2-like phenotype of macrophages, we performed a rescue experiment by transfecting C1qbp-knockdown cells with plasmids expressing full-length or truncated C1qbp and then co-culturing them with 4T1 cells (Figure S6B). Re-expression of the full-length C1qbp restored and even slightly increased the expression of M2-associated marker Arg1, as compared to the vector control.

Meanwhile, the ability of truncated C1qbp lacking the Vtn binding domain to restore Arg1 expression in C1qbp-knockdown cells was significantly decreased as compared to the full-length C1qbp (Figure S6C), indicating that the Vtn-C1qbp interaction is required for Arg1 expression and for shifting macrophages towards the M2-like phenotype. In contrast, neither the full-length nor the truncated C1qbp failed to suppress the increased Inos expression in C1qbp-knockdown cells (Figure S6D). It is possible that loss of C1qbp might have caused an irreversible change in the M1-like phenotype, or at least in the expression of some M1 markers.

### Vtn-C1qbp mediates CD16-dependent Shp1 recruitment in macrophage cells

**Figure 6:**
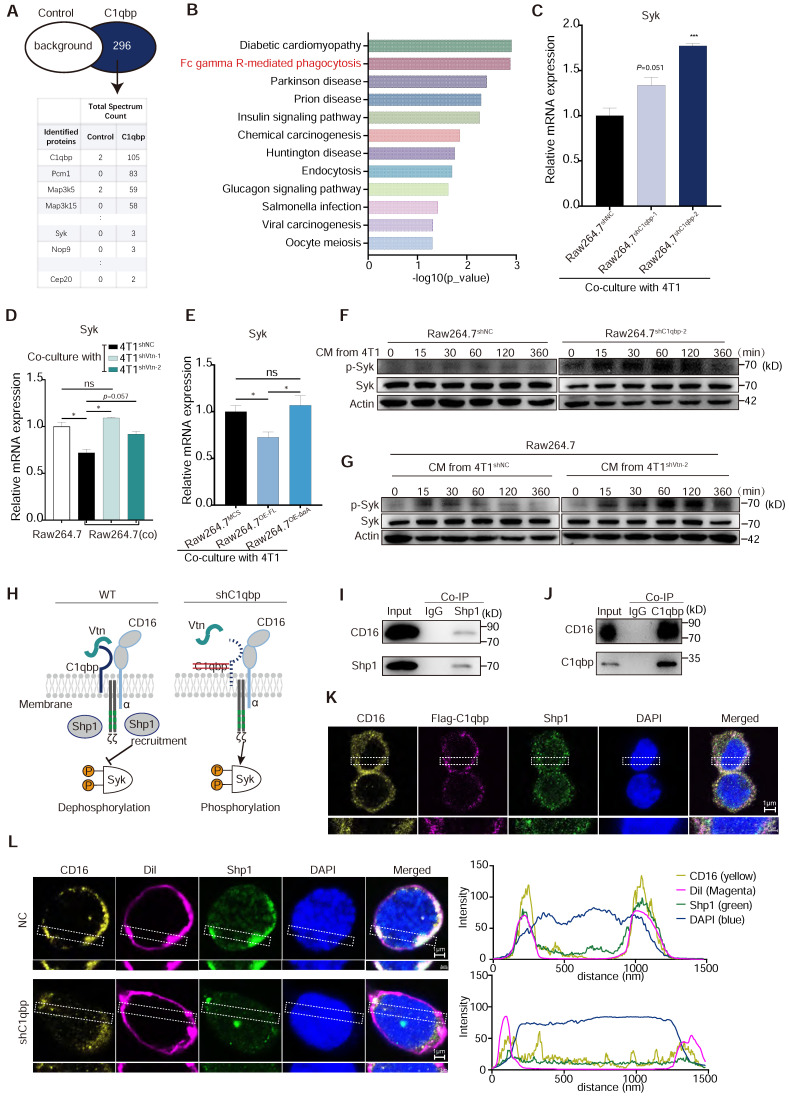
** Vtn-C1qbp inhibits Syk phosphorylation via Shp1. (A)** Mass spectrum showing C1qbp interactive proteins. C1qbp-IP was conducted in Raw264.7 cells. Mass spectrum was subsequently performed in the C1qbp-IP proteins. Control, IgG-IP proteins from Raw264.7. **(B)** KEGG pathway enrichment analysis of the unique 296 proteins in C1qbp-IP proteins compared to control (A). **(C)** qRT-PCR analysis of Syk gene in control and C1qbp-knockdown Raw264.7 cells co-culture with 4T1 cells. **(D)** qRT-PCR analysis of Syk gene in wildtype Raw264.7 and control and C1qbp-knockdown Raw264.7 cells co-culture with Vtn knockdown or control 4T1 cells for 48 h. **(E)** qRT-PCR analysis of Syk genes in the Full-length C1qbp (Raw264.7^OE-FL^), mutant-C1qbp (Raw264.7^OE-ΔαA^) overexpression and control Raw264.7 (Raw264.7^MCS^) co-culture with 4T1 cells for 48 h. **(F)** Western blot analyses of Syk, p-Syk in whole-cell lysates of control or C1qbp-knockdown Raw264.7 cells treated with CM from 4T1 cells for the indicated time. **(G)** Western blot analyses of Syk, p-Syk in whole-cell lysates of Raw264.7 cells treated with CM from control or Vtn knockdown 4T1 cells for the indicated time. p-Syk: phosphorylation of Syk. **(H)** A model summarizing the role of Vtn-C1qbp in Syk phosphorylation. Schematic drawing of CD16 showing the two Ig-like extracellular subunits and the α-chain (blue) in complex with CD3ζ (grey) chains. Immunoreceptor tyrosine-based activation motifs (ITAMs) motifs are shown in green. **(I and J)** Co-immunoprecipitation of Shp1 and CD16 (I) or C1qbp and CD16 (J) in Raw264.7 stimulated with conditioned medium (CM) of 4T1 cells. **(K)** Immunofluorescence staining of CD16, flag-C1qbp, and Shp1 in flag-C1qbp expressed Raw264.7 cells. The CD16, flag-C1qbp, Shp1, and nucleus are stained yellow, magenta, green, and blue (DAPI), respectively. Scale bars, 1 μm. **(L)** Immunofluorescence staining of CD16, cell surface (1,1'-Dioctadecyl-3,3,3',3'-tetramethylindocarbocyanine perchlorate, Dil) and Shp1 in Raw264.7 cells. The CD16, cell surface, Shp1, and nucleus are stained yellow, magenta (Dil), green, and blue (DAPI), respectively. Scale bars, 1 μm. The graphs (right) show averaged fluorescent intensity along the regions of interest indicated on the overlay images.

To investigate how C1qbp affects macrophage phagocytosis, we used IP-coupled mass spectrometry analysis to identify the interacting proteins of C1qbp. C1qbp was immunoprecipitated from Raw264.7 cells using an anti-C1qbp antibody, and its interaction proteins were identified by mass spectrometry. We detected 296 specific binding proteins of C1qbp (Figure 6A). KEGG pathway enrichment analysis revealed that they were mainly enriched in the Fcγ receptor-mediated phagocytosis and the phagocytosis-related, endocytosis pathway (Figure 6B). Meanwhile, Syk, an essential effector of Fcγ-mediated phagocytosis and macrophage activation, was identified among the C1qbp-specific binding proteins (Figure 6A). Therefore, we further investigated the effect of C1qbp on Syk expression and phosphorylation. C1qbp-knockdown Raw264.7 cells co-cultured with 4T1 cells had higher expression levels of Syk as compared to the control Raw264.7 cells (Figure 6C). We next determined the role of the Vtn-C1qbp signaling, which suppresses macrophage phagocytosis, in Syk protein expression and phosphorylation in macrophages. Raw264.7 cells co-cultured with 4T1 cells had decreased Syk expression as compared to the Raw264.7 not co-cultured with 4T1, while co-culture with Vtn-knockdown 4T1 cells significantly increased Syk expression to approximately the same level as in the Raw264.7 cells not co-cultured with 4T1 (Figure 6D). To further demonstrate the effect of Vtn-C1qbp signaling on the expression of Syk, we constructed Raw264.7 cells overexpressing full-length and truncated C1qbp. Overexpression of full-length C1qbp, but not truncated C1qbp lacking the Vtn binding domain, in Raw264.7 cells co-cultured with 4T1 cells significantly attenuated Syk expression as compared to control Raw264.7 cells expressing the vector (Figure 6E), indicating that the Vtn-C1qbp signaling inhibits Syk expression in macrophages. Moreover, silencing Vtn in 4T1 cells did not further increase the enhancement of Syk expression by C1qbp-knockdown in macrophages (Figure S7A), suggesting that the effect of Vtn on Syk expression depends on C1qbp. Furthermore, Syk was rapidly phosphorylated in Raw264.7 cells in response to treatment with conditioned medium (CM) from 4T1, which occurred at 15 min after treatment and persisted for about 2 h; and 4T1 CM-induced Syk phosphorylation was enhanced by C1qbp-knockdown in Raw264.7 cells (Figure 6F and Figure S7B). Meanwhile, CM from Vtn-knockdown 4T1 cells induced augmented phosphorylation of Syk in Raw264.7 cells as compared to CM from the control 4T1 cells (Figure 6G and Figure S7C). These results demonstrate that the Vtn-C1qbp signaling suppresses Syk phosphorylation in macrophages.

We further investigated the mechanism and downstream molecules underlying the impact of C1qbp on Syk phosphorylation. As a protein located in lipid rafts, C1qbp can exert its function by interacting with receptors present on the cellular membrane 30. In our IP-mass spectrometry analysis, we identified several proteins that interact with C1qbp. Notably, these included the CD16 receptor and the tyrosine phosphatase Src homologous region 2 containing phosphatase 1 (Shp1), which is encoded by the gene Ptpn6. Shp1 has been implicated in Syk phosphorylation 31. Shp1 plays a crucial role in regulating tyrosine phosphorylation. It can be targeted by binding to phosphorylated immune receptor tyrosine-based inhibitory motifs (ITIM) and subsequently act in conjunction with ITIM-containing molecules to restrain signaling pathways, including those involved in Syk phosphorylation 32. The CD16 receptor contains ITAM motifs and was reported to inhibit macrophage phagocytosis of E. coli by recruiting Shp1 to the membrane during E. coli-FcγRIII interaction 33. Based on these previous findings, we hypothesize that Vtn-C1qbp facilitates CD16-induced Shp1 recruitment, and that consequently, Shp1 recruitment is reduced by knockdown of Vtn or C1qbp (Figure 6H).

To investigate the interactions among C1qbp, CD16, and Shp1, we conducted immunoprecipitation-coupled Western blotting analysis. We found that CD16 co-immunoprecipitated Shp1 and with C1qbp in Raw264.7 cells treated with 4T1 CM (Figure 6I-J). Results from immunofluorescence staining revealed a partial co-localization of CD16, C1qbp, and Shp1 on the cell surface, which was further confirmed by the fluorescence intensity profiles along a rectangular area (white dotted line) crossing the nucleus (Figure 6K and Figure S8A). To explore whether C1qbp influences the recruitment of Shp1 to CD16, we performed Shp1 staining in C1qbp-knockdown Raw264.7 cells. In control cells, Shp1 protein was prominently recruited to the plasma membrane and partially co-localized with CD16. In contrast, after C1qbp knockdown, the co-localization of CD16 with Shp1 on the plasma membrane was significantly diminished (Figure 6L). This observation was also corroborated by subsequent fluorescence intensity analysis. Based on the experimental results, we propose that the reduction in Syk phosphorylation may be resulted from C1qbp-mediated recruitment of Shp1, which de-phosphorylates Syk. In addition, we have also observed a decrease in the expression of CD16 and Shp1, which could also potentially contribute to the increase of Syk phosphorylation.

We further examined the expression of genes functioning downstream of Syk in Fcγ-receptor-mediated phagocytosis, including Pi3k, Akt, Dynamin2, Plcg1, Arf6, Vav1, and Pla2g4a. Raw264.7 co-cultured with Vtn-knockdown 4T1 cells displayed significantly increased Vav1 and Pla2g4a expression as compared to those co-cultured with the control 4T1 cell, while Vtn-knockdown in 4T1 cells had no effect on the other Syk downstream genes including Pi3k, Akt, Dynamin2, Plcg1, and Arf6 (Figure S8B). We also examined the effect of C1qbp on Vav1 and Pla2g4a. C1qbp-knockdown Raw264.7 cells co-cultured with 4T1 cells expressed increased levels of Vav1 but decreased levels of Pla2g4a as compared to the control Raw264.7 cells (Figure S8C). Moreover, overexpression of the full-length C1qbp, but not the truncated C1qbp lacking the Vtn binding domain, in Raw264.7 cells co-cultured with 4T1 cells significantly reduced Vav1 expression as compared to the control Raw264.7 cells, while both full length and truncated C1qbp had only minimum effect of the expression of Pla2g4a (Figure S8D). Taken together, these results suggest that the Vtn-C1qbp signaling suppresses phagocytosis via Syk and its downstream effector Vav1.

### Vtn silencing and the anti-CD47 antibody synergize to mediate anti-tumor activity and augment TAM expansion and phagocytosis

**Figure 7:**
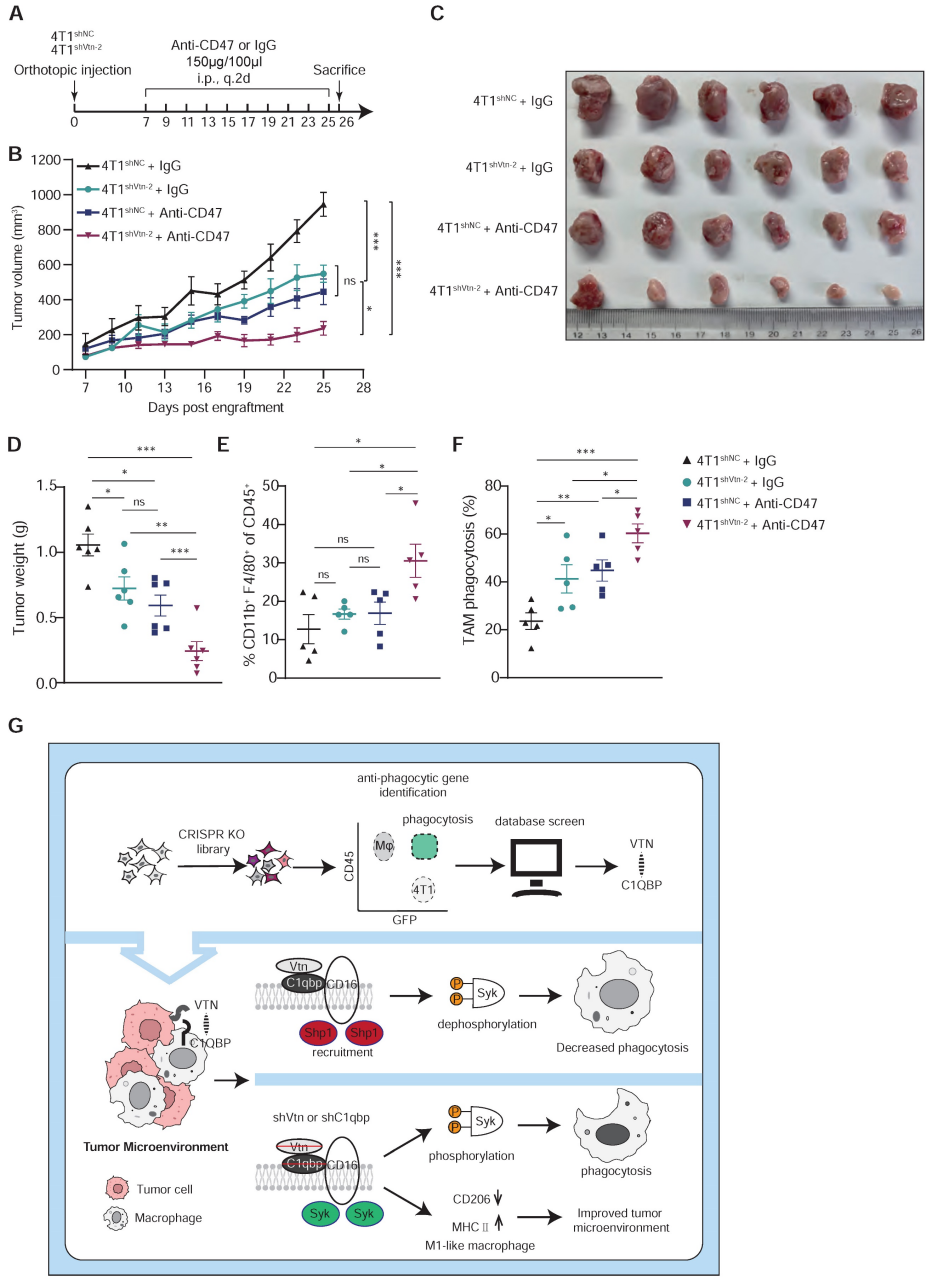
** Vtn silencing synergizes with CD47 blockade to increase cancer cell susceptibility to phagocytosis. (A)** Schematic diagram of the mouse experiment (6 mice in each group). **(B to D)** Tumor volumes (B), tumor weights at end of experiments (C), and representative images (D) were measured (n = 6, mean ± SEM, ns not significant, *P < 0.05, **P < 0.01, ***P < 0.001). **(E)** TAM phagocytosis is assessed as the frequency of CD11b^+^F4/80^+^GFP^+^ events out of total CD11b^+^F4/80^+^events. Numbers indicate frequency of phagocytosis events out of all macrophages. Plots are representative of three experimental replicates. *p < 0.05, **p < 0.01, ***p < 0.001, by unpaired two-sided Student's t-test. **(F)** TAM populations from mouse experiment were analyzed by FACS. n = 5. **(G)** Schematics summarizing the Vtn-C1qbp signaling in regulating macrophage phagocytosis and phenotype.

CD47 is the most studied "don't eat me" signal that is overexpressed on tumor cells and inhibits macrophage activity through interaction with its receptor SIRPα. CD47 blockade has shown promising clinical activity in early human trials. Additionally, our analysis of public single-cell sequencing data revealed that C1qbp-positive TAMs exhibit more immunosuppressive interactions with tumor cells compared to C1qbp-negative TAMs in TNBC patients, including CD47-SIRPα (Figure S9A-C). Based on the previously reported role of Vtn-C1qbp signaling in tumor immune response, we further investigated whether Vtn knockdown and anti-CD47 antibody treatment have a synergistic anti-tumor effect. We inoculated control and Vtn knockdown breast cancer cells into mouse fat pads, treated the mice with IgG control or an anti-CD47 antibody every other day from day 7 to day 25, and measured tumor sizes (Figure 7A). As compared to the untreated group, either Vtn knockdown or the anti-CD47 antibody treatment alone reduced tumor growth and tumor weight to similar levels, while the anti-CD47 antibody treatment in mice bearing Vtn-knockdown tumors led to an even stronger suppression of tumor growth and tumor weight (Figure 7B-D). Enhanced tumor inhibition by the combination of Vtn-knockdown and anti-CD47 antibody treatment correlated with an increase in the infiltration of macrophages to the tumor microenvironment relative to no treatment or either treatment alone (Figure 7E). Consistently, Vtn knockdown or treatment with the anti-CD47 antibody alone in 4T1 cells promoted phagocytic clearance by macrophage, as expected, while combination of Vtn-knockdown and the anti-CD47 mAb treatment showed a further increase in phagocytosis by macrophages (Figure 7F), demonstrating a potential synergy between Vtn-knockdown and the anti-CD47 antibody.

To further determine whether macrophages are required for tumor reduction in Vtn knockdown, we used clodronate liposomes to deplete macrophages (Figure S10A). We then compared the effects of macrophage depletion on Vtn-knockdown tumor-bearing mice (Figure S10B). Notably, the tumor suppressive efficacy of Vtn knockdown was attenuated in the macrophage depletion group (Figure S10C-D), which was significantly larger than that in the Vtn knockdown group. This suggests that macrophages play a major role in tumor suppression by Vtn knockdown.

In conclusion, using a CRISPR/Cas9 knockout library screen targeting tumor cells and macrophages respectively in a phagocytosis assay, we identified an interaction between Vtn expressed in cancer cells and C1qbp expressed on the plasma membrane of tumor-associated macrophages, which constrains macrophage phagocytosis and shifts macrophages more skewed toward the M2-like phenotype. Vtn binding to C1qbp on macrophage plasma membranes facilitated CD16-mediated Shp1 recruitment, thereby reducing Syk phosphorylation, which in turn inhibits macrophage phagocytosis (Figure 7G). This work may provide a new molecular target for the treatment of cancer.

## Discussion

Macrophage phagocytosis of tumor cells is regulated by a variety of pro-phagocytosis (eat me) and anti-phagocytosis (don't eat me) signals, particularly through ligand-receptor interactions at the cell-cell interface 2. CD47 was reported to be highly expressed on TNBC tumors and its expression was correlated with worse patient prognosis and outcomes 34. However, there is a study demonstrated that a lower phagocytosis rate was observed of MDA-MB-231 (TNBC) cells to CD47-blocking antibody as compared to that of Raji cells (non-Hodgkin's lymphoma), suggesting that other signals inhibiting phagocytosis exist between TNBC and macrophage 35. Based on the above background, we performed CRISPR library screens to search for genes that inhibit phagocytosis, which led to the identification of the Vtn-C1qbp ligand-receptor pair that regulates macrophage phagocytosis.

In previous studies, the interaction between Vtn and C1qbp was detected but poorly characterized. Our co-immunoprecipitation experiments revealed for the first time that Vtn secreted by tumor cells could bind C1qbp on macrophage plasma membranes. Moreover, the binding of Vtn to C1qbp was significantly decreased after truncating the first 20 amino acids of the mature form of C1qbp. Rescue studies using the truncated form of C1qbp lacking the Vtn binding domain further demonstrated that Vtn exerts an inhibitory effect on macrophage phagocytosis by binding to C1qbp on the macrophage plasm membrane. Previous studies on ligand-receptor pairs, such as CD24-Siglec10 and CD47-Sirpα 6, 36, used neutralizing antibodies to directly verify their effects on phagocytosis. However, small molecule inhibitors or antibodies that could block C1qbp are not currently available to directly validate our results. Therefore, our future studies will be directed to the discovery of small molecule inhibitors and generation of neutralizing antibodies for C1qbp.

Moreover, Vtn knockdown led to a significant increase in M1-like macrophages as well as CD8^+^ T cell activation in the tumor microenvironment, suggesting that the enhanced phagocytosis induced an indirect activation of adaptive immunity and thus decelerates tumor growth. It has also been reported that Vtn plays a key role in thrombosis, wound healing, and tissue repair 37, the processes in which M2-type macrophages are major participants 38. Consistently, we also found in this study that the release of Vtn from tumor cells induces macrophages to favor the M2-like, which indicates that Vtn is also a crucial mediator of macrophage polarization in the tumor microenvironment.

C1qbp was first identified in the Raji cell plasma membrane, but subsequent studies found that it can also be localized intracellularly or in mitochondria 28. This also coincides with our immunofluorescence results. The main function of C1qbp in mitochondria is to sustain oxidative phosphorylation, which plays a pivotal role in tissue maintenance and normal function 39. Since phagocytosis is closely correlated with oxidative phosphorylation 40, the intracellular form of C1qbp may also play a role in phagocytosis. We will explore this possibility in our future studies. Our results showed that after knockdown of C1qbp, macrophages co-cultured with tumor cells significantly preferred the M1-like type. This finding indicates that C1qbp is crucial for the conversion of macrophage phenotype and thus represents a promising target for immunotherapy.

The membrane form of C1qbp is currently reported to play a role in viral infection, and it is recognized as an essential pathogen recognition receptor. The core protein of hepatitis C virus (HCV) and the envelope glycoprotein of the human immunodeficiency virus (HIV) gp41 can employ C1qbp to target CD4^+^ T cells or monocytes to mediate immunosuppression 41, 42. However, the role of the membrane form of C1qbp in cancer development has seldomly been reported. Intriguingly, we found that the membrane form of C1qbp was only present in CD45-positive cells, not in tumor cells, and was predominantly distributed on macrophages. Furthermore, we noticed that almost all macrophages infiltrated into 4T1 tumors expressed the membrane form of C1qbp, suggesting that C1qbp plays an important role in macrophages. Meanwhile, at the resting condition of macrophages, C1qbp was rarely detected on macrophage plasma membranes, but stimulation with tumor supernatant or co-culture with tumor cells significantly enhanced the transfer of C1qbp to the plasma membrane in macrophages. We propose that there is a distinct mechanism regulating the transfer of C1qbp to the plasma membrane, potentially influenced by specific factors secreted by the tumor. Such a mechanism may share similarities with the translocation of C1qbp in pancreatic cancer tumor cells 43.

CD16, also known as FcγRIIIA, has been widely reported to be associated with Syk activation. In sepsis, CD16 interacts with E. coli induces Shp1 recruitment and phosphatidylinositol-3 kinase (PI3K) dephosphorylation, which inhibits E. coli phagocytosis. Since results from our mass spectrometry analysis revealed both CD16 and Shp1 as C1qbp interacting proteins, we investigated whether C1qbp had an effect on Shp1 recruitment. Immunofluorescence co-localization analysis demonstrated that C1qbp increased Shp1 recruitment and suggested that CD16 may have a dual role in macrophage phagocytosis.

Based on these results, we further assessed whether knockdown Vtn had a synergistic effect with the anti-CD47 antibody treatment on tumor growth inhibition. The results showed that the combination of Vtn knockdown and treatment with the anti-CD47 antibody was highly effective in suppressing 4T1 tumors and elevating macrophage infiltration. Furthermore, macrophage phagocytosis was significantly increased by the combinatorial treatment as compared to the individual treatments, indicating a potential synergistic effect of inhibition of both Vtn and CD47. Our studies have thus provided proof of principle for a novel therapeutic strategy for cancer by targeting the tumor microenvironment.

## Supplementary Material

Supplementary figures and table.

## Abbreviations

B2M: beta-2-microglobulin
BMCs: bone marrow cells
BMDMs: bone marrow-derived macrophages
C1qbp: complement C1Q binding protein
CM: conditional medium
DAB: 3,30-diaminobenzidine
DAPI: 40,6-diamidino-2-phenylindole
DC: dendritic cells
DMEM: dulbecco's modified Eagle's medium
FACS: fluorescence activated cell sorter
Fzd2: frizzled Class Receptor 2
GEO: gene expression omnibus
GDC: genomic data commons
GFP: green fluorescent protein
H&E: hematoxylin, and eosin
HCV: hepatitis C virus
HIV: human immunodeficiency virus
IHC: immunohistochemical
IP: immunoprecipitation
Kdr: kinase insert domain receptor
MDSCs: marrow-derived suppressor cells
MS: mass spectrometry
MAGeCK: model-based analysis of genome-wide CRISPR-Cas9 knockout
MOI: multiplicity of infection
PM: peritoneal macrophage
PBS: phosphate buffered saline
PI3K: phosphatidylinositol-3 kinase
ITIM: phosphorylated immune receptor tyrosine-based inhibitory motifs
PVDF: polyvinylidene fluoride
RIPA: radioimmunoprecipitation assay
RRA: robust rank aggregation
SpM: splenic macrophage
TCGA: the cancer genome atlas
TNBC: triple-negative breast cancer
TIMER: tumor immune estimation resource
TAMs: tumor-associated macrophages
Shp1: tyrosine phosphatase Src homologous region 2 containing phosphatase 1
Vtn: vitronectin
