# Supplementary material for: Targeting tumor cell-to-macrophage communication by blocking Vtn-C1qbp interaction inhibits tumor progression via enhancing macrophage phagocytosis
Source: Theranostics. 2024 Apr 22;14(7):2757–76. doi: 10.7150/thno.94537 (PMC11103506; doi:10.7150/thno.94537)
Supplement: Supplementary file 1 — Supplementary figures and table. [file thnov14p2757s1.pdf]

Supplementary Materials

Fig.S1

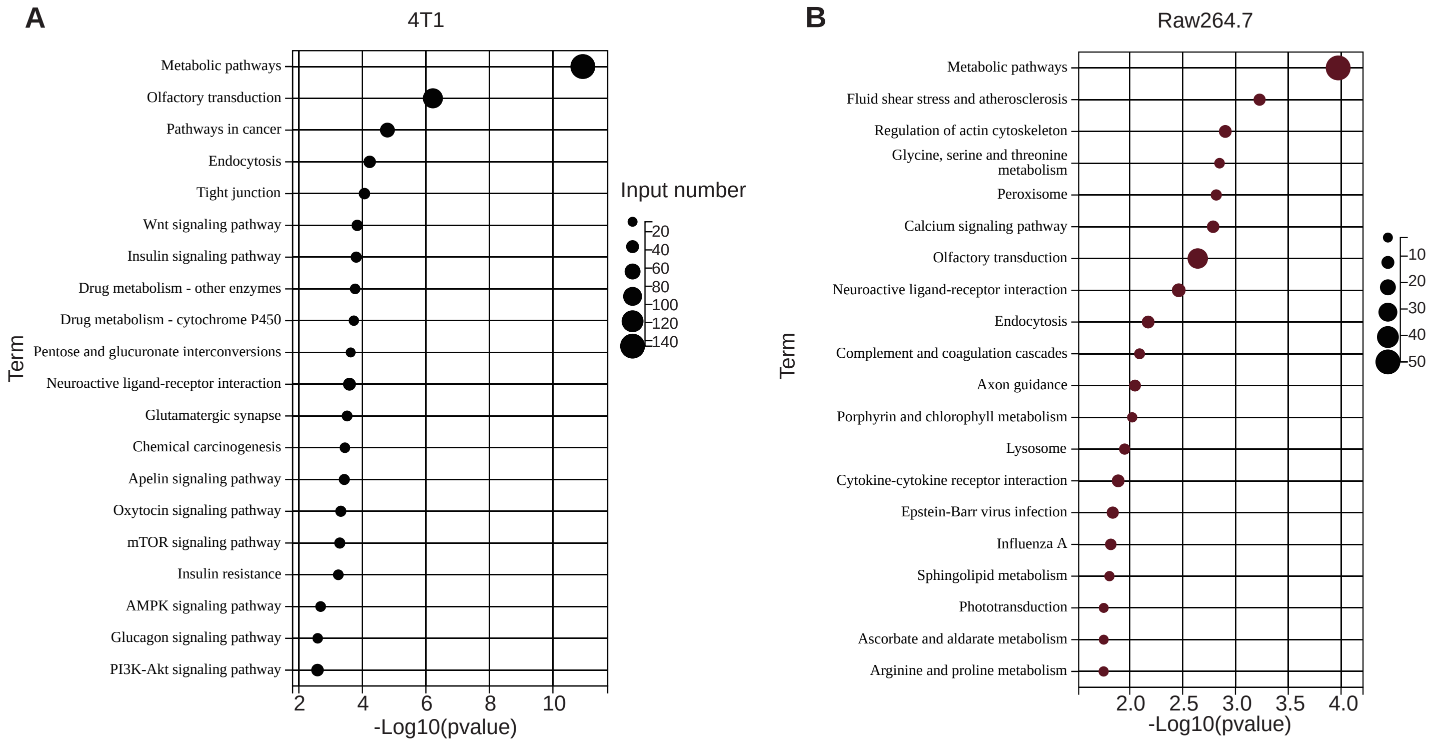

Fig. S1 The pathways enrichment for candidate genes

(A and B) KEGG pathway enrichment analysis of positive sgRNA hits in 4T1 (A) and positive sgRNA hits in Raw264.7 (B).

Fig.S2

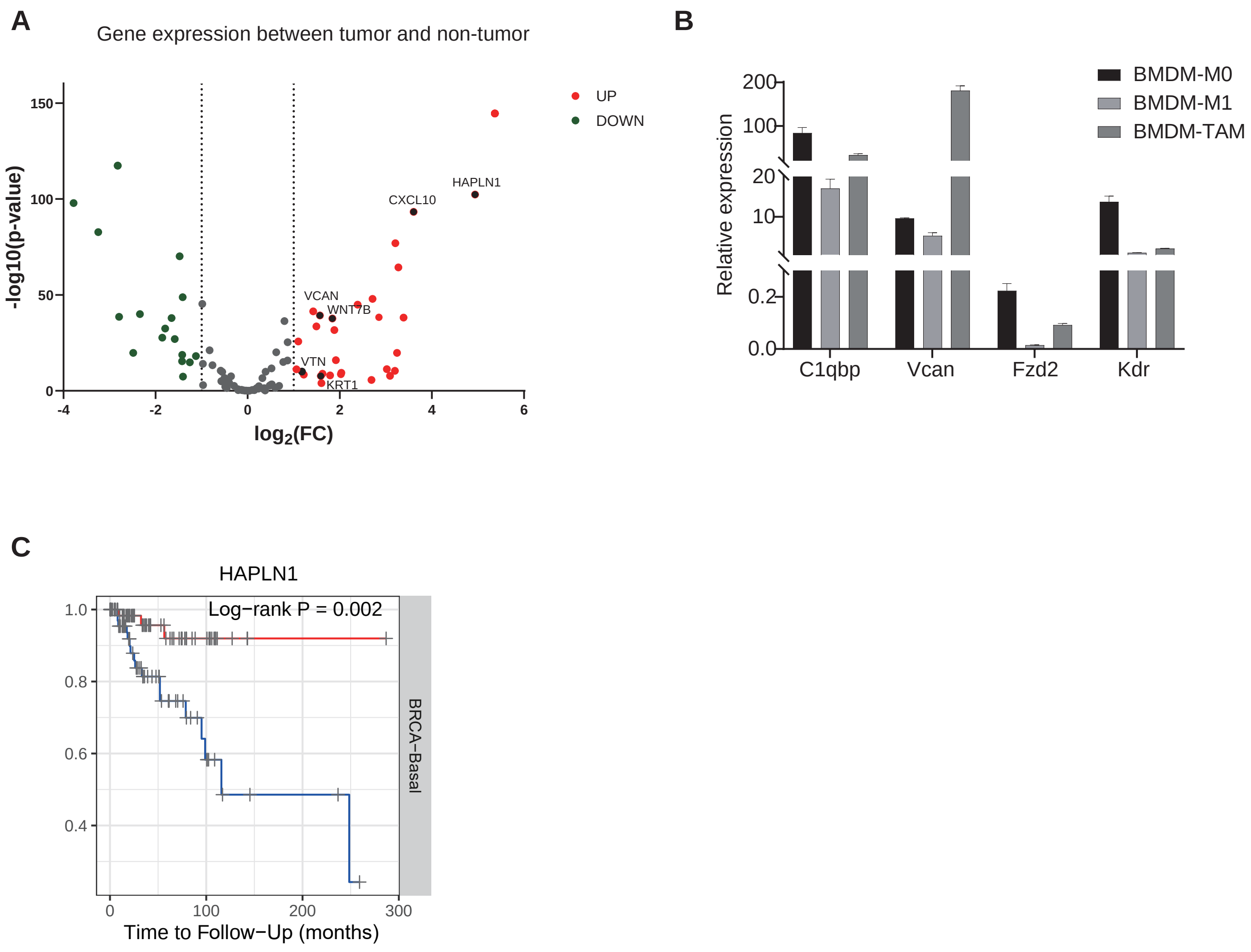

Fig. S2 Analysis of the expression of candidate genes in tumor and normal tissues and the effect on survival

(A) The differentially expressed of screened indicated inhibitory genes were identified from The Cancer Genome Atlas. **(B)** qRT-PCR analysis of Raw264.7 candidate genes in BMDM-M0 (unstimulated), BMDM-M1 (stimulated with LPS), and BMDM-TAM (stimulated with conditioned medium of 4T1 cells). **(C)** Overall survival of TCGA patients with basal breast cancer(n=140) with high or low Hapln1 expression as defined by the median. Two-sided P value computed by a log-rank (Mantel-Cox) test.

Fig.S3

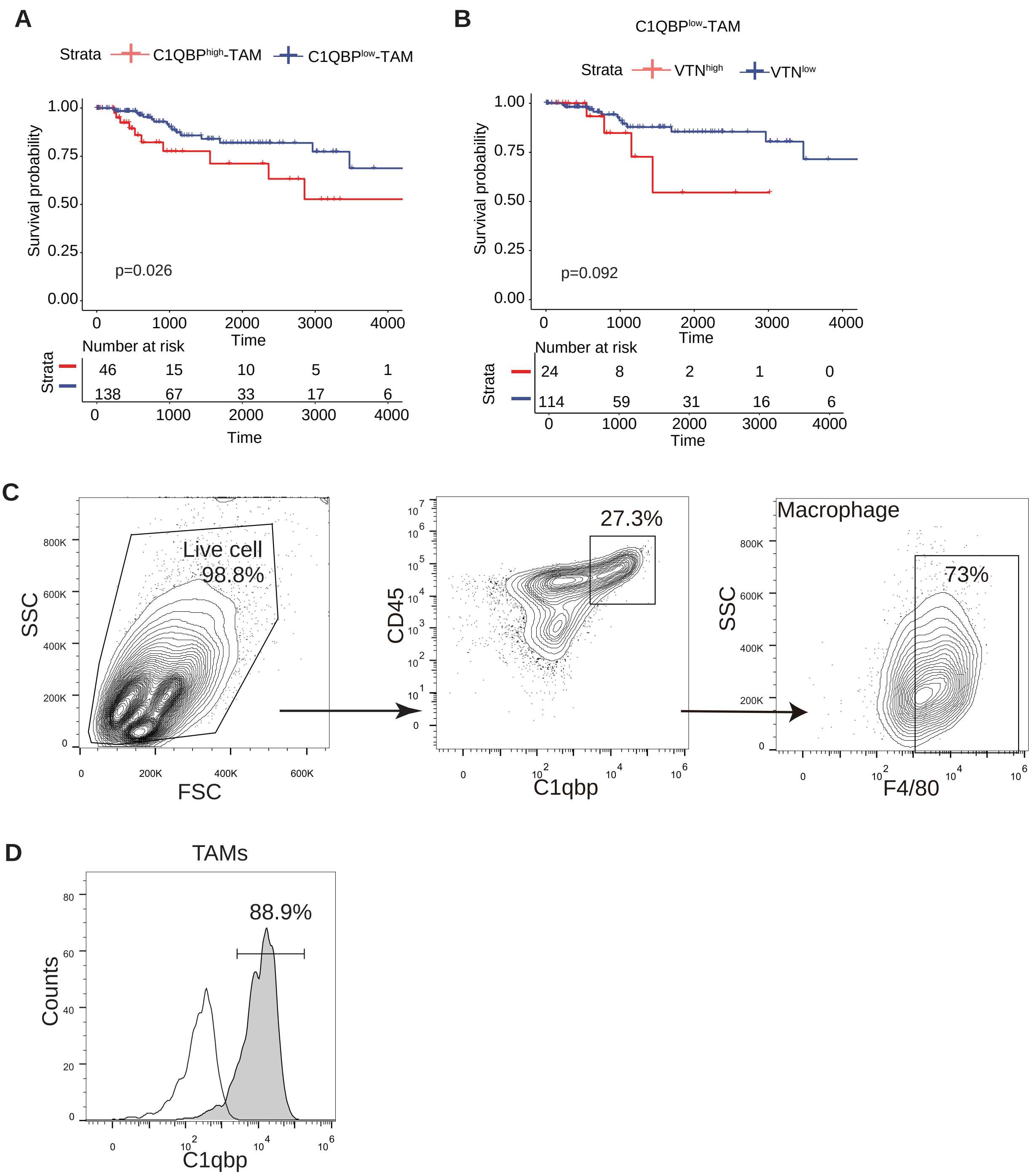
A
Strata C1QBP^high-TAM C1QBP^low-TAM
Survival probability
p=0.026
Time
Number at risk
Strata
46 15 10 5 1
138 67 33 17 6
B
C1QBP^low-TAM
Strata VTN^high VTN^low
p=0.092
24 8 2 1 0
114 59 31 16 6
C
Live cell
98.8%
SSC
FSC
27.3%
CD45
C1qbp
Macrophage
73%
F4/80
D
TAMs
88.9%
Counts
C1qbp

Fig. S3 The survival analysis of VTN expression and $C1QBP^{+}$ macrophage in TCGA TNBC patients

(A and B) Survival curves comparing $C1QBP^{high}$ macrophage infiltration patients(n=46) or $C1QBP^{low}$ macrophage infiltration patients(n=184) (A), and high or low expression of VTN in $C1QBP^{low}$ macrophage infiltration patients(n=138) (B) in TCGA-TNBC database. Two-sided P value computed by a log-rank (Mantel-Cox) test. Numbers of subjects at risk in the high group (red) compared with the other group (blue) are indicated below the x-axes. **(C)** Gating strategy for $CD45^{+}$ and $C1qbp^{+}$ cells among 4T1 tumors. TAMs in $CD45^{+}C1qbp^{+}$ were assessed as the frequency of $F4/80^{+}$ events out of total $CD45^{+}C1qbp^{+}$events. **(D)** Representative histogram measuring the expression of C1qbp by 4T1 tumor-bearing mouse TAMs.

Fig.S4

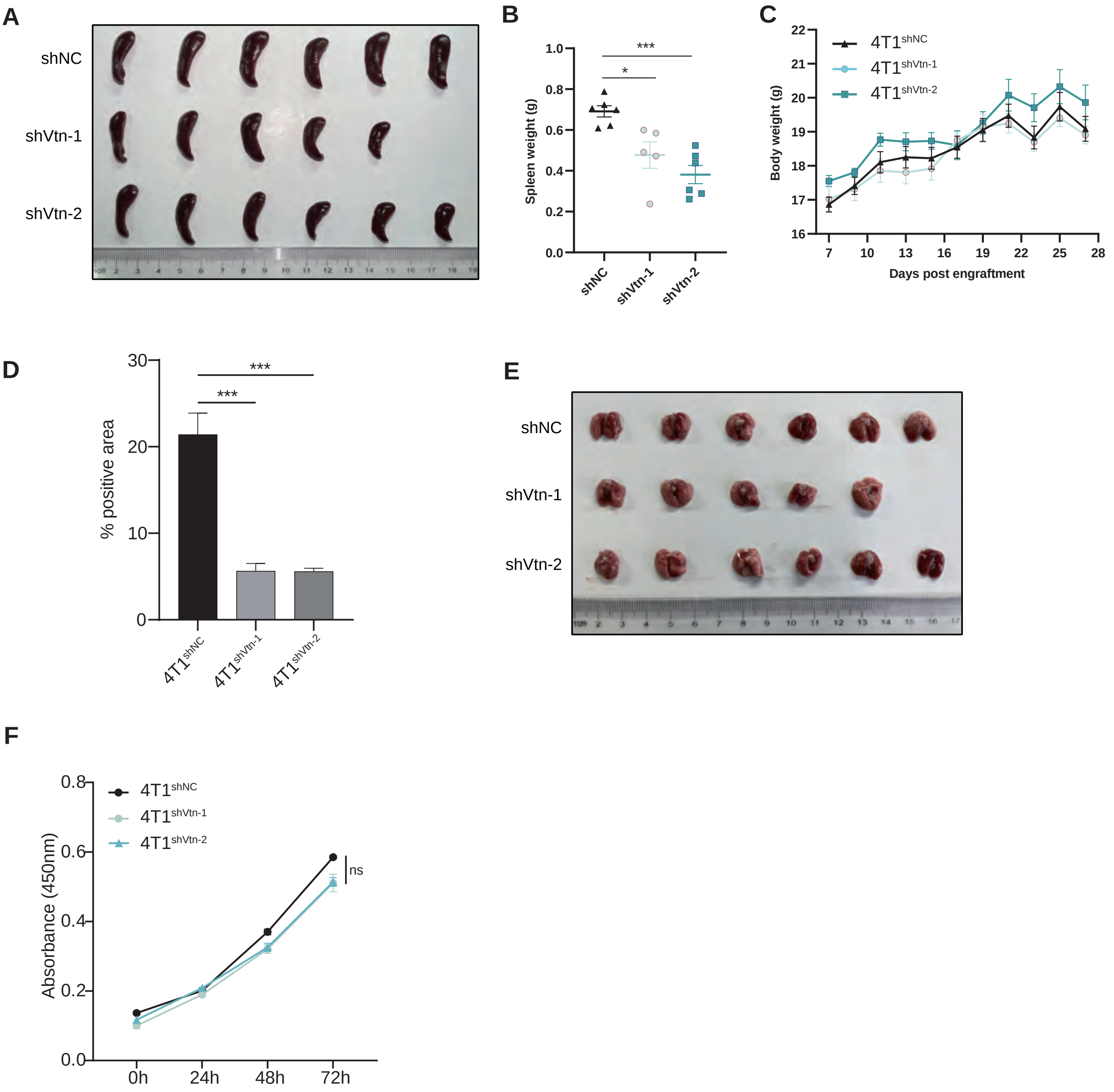

Fig. S4 Vtn-knockdown significantly reduced the size and weight of spleen in 4T1 tumor-bearing mice

(A) Representative images of spleen dissected from mice 26 days post-inoculation of 4T1 cells. **(B)** Graph shows the mean spleen weight ± SEM of control and Vtn-knockdown tumor-bearing mice. **(C)** The body weight changes of the mice received indicated treatments. **(D)** The % of Vtn positive area was compared between control and Vtn-knockdown tumor. **(E)** Representative images of lungs dissected from mice 26 days post-inoculation of 4T1 cells. **(F)** The growth curve of control and Vtn-knockdown cells. Each of the experiments was repeated 5 times, n=5.

Fig.S5

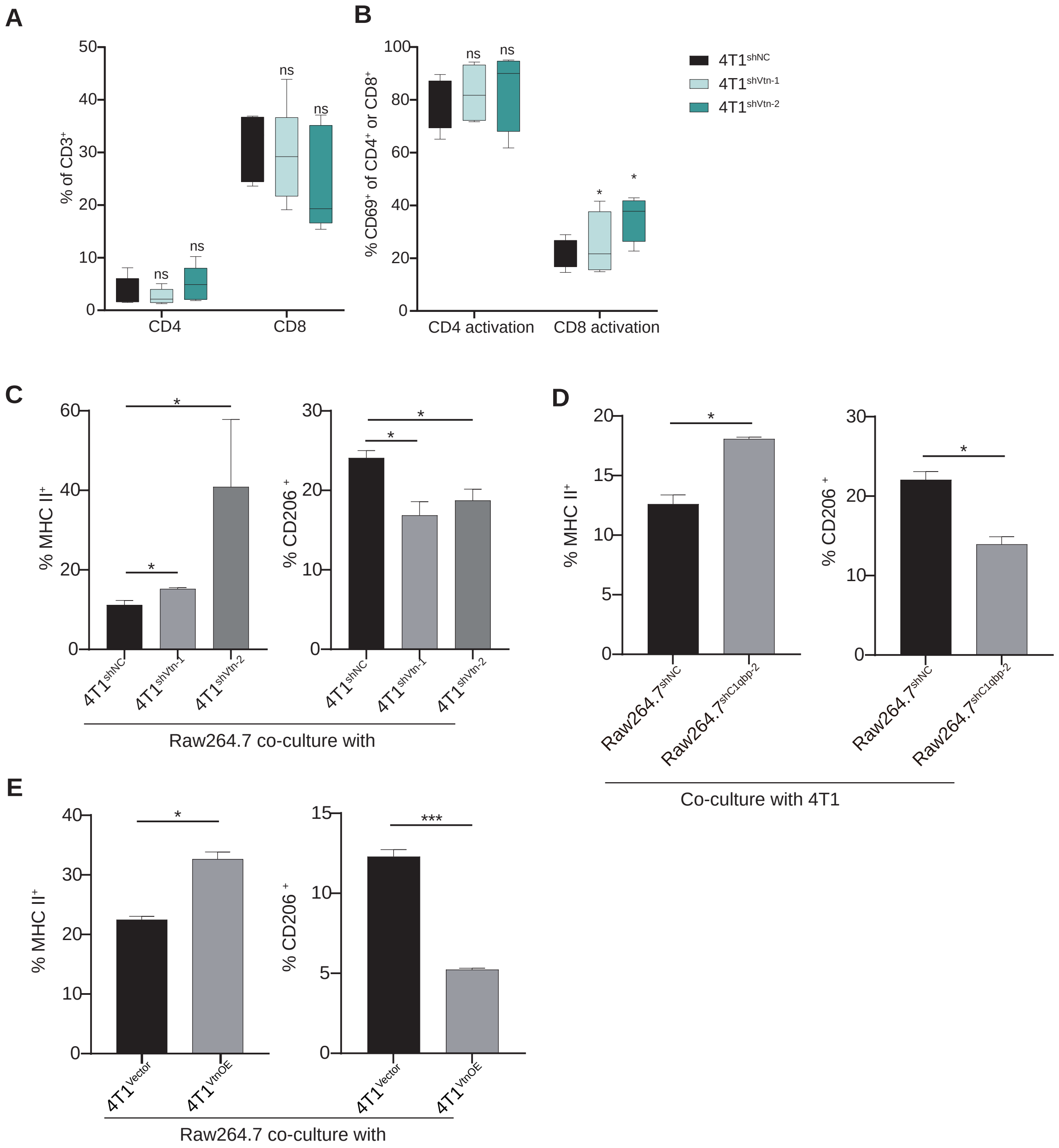

Fig. S5 Knockdown of Vtn induces activation of CD8$^+$ T cell in tumor and polarized macrophage toward M1-like phenotype in vitro

(A and B) Box plots show the percent of CD4$^+$ and CD8$^+$ T cells in T lymphocytes(CD3$^+$) (A), the percent of CD69$^+$ T cells in CD4$^+$ or CD8$^+$ T cells (B). **(C to E)** Quantitative analysis of M1-like (MHCII$^+$) and M2-like (CD206$^+$) subpopulations in Raw264.7 cells co-culture with Vtn knockdown or control 4T1 cells (C), with Vtn-overexpression or control (Vector) 4T1 cells (E) and in control and C1qbp knockdown Raw264.7 cells co-culture with 4T1 cells (D) for 48 h.

Fig.S6

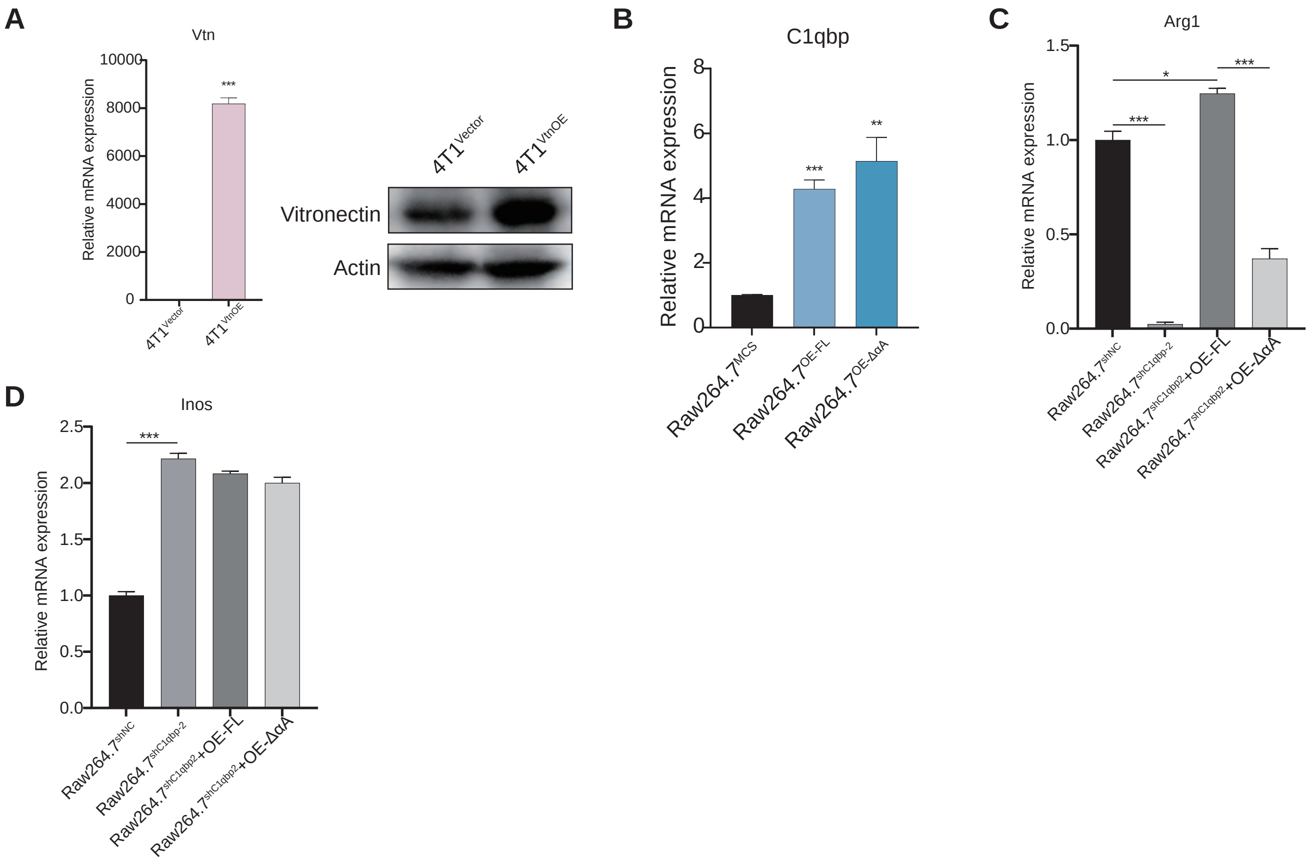

Fig. S6 Overexpression of Vtn and full-length or truncated C1qbp in 4T1 and Raw264.7 respectively and its effect on macrophage phenotype

(A) qRT-PCR and Western blot analysis of Vtn in Vtn or empty vector-transfected 4T1 cells.Raw264.7 cells overexpressing full-length C1qbp and C1qbp truncates. **(B)** qRT-PCR analysis of full-length C1qbp and C1qbp truncates overexpression Raw264.7 cells. **(C and D)** qRT-PCR analysis of macrophage marker gene Arg1 (C) and Inos (D) in control, C1qbp-knockdown Raw264.7 and C1qbp-knockdown Raw264.7 transfected with vector expressing full-length C1qbp or truncated C1qbp co-culture with 4T1 cells for 48 h.

Fig.S7

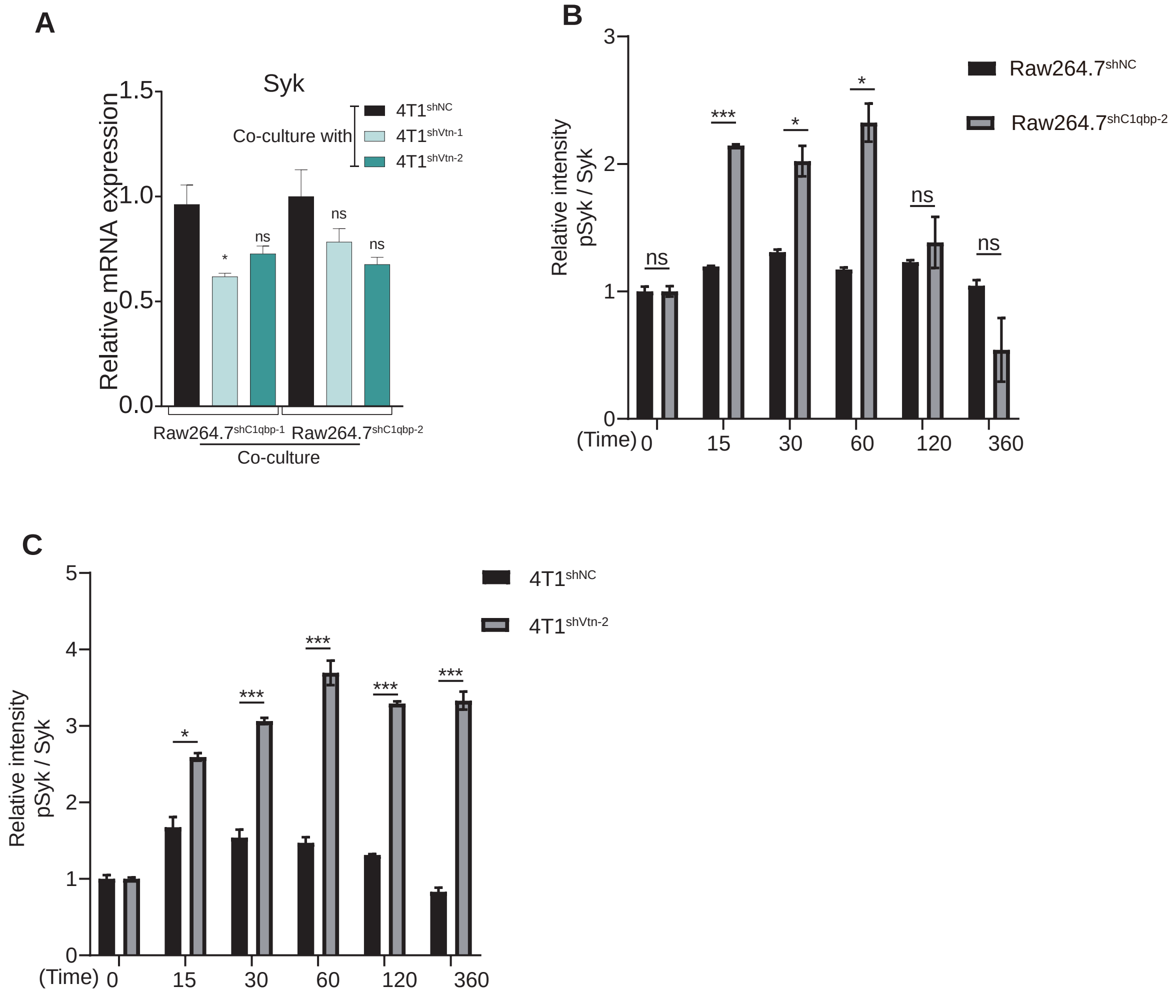

Fig. S7 Silencing Vtn did not further increase the Syk expression after C1qbp-knockdown

(A) qRT-PCR analysis of Syk gene in C1qbp-knockdown Raw264.7 co-culture with Vtn knockdown or control 4T1 cells for 48 h. **(B and C)** The histogram represents the quantification of P-Syk /total Syk ratios in control or C1qbp-knockdown Raw264.7 cells treated with CM from 4T1 cells (B) and in Raw264.7 cells treated with CM from control or Vtn knockdown 4T1 cells (C). Data are represented as means ± SEM and expressed relative to Time 0.

Fig.S8

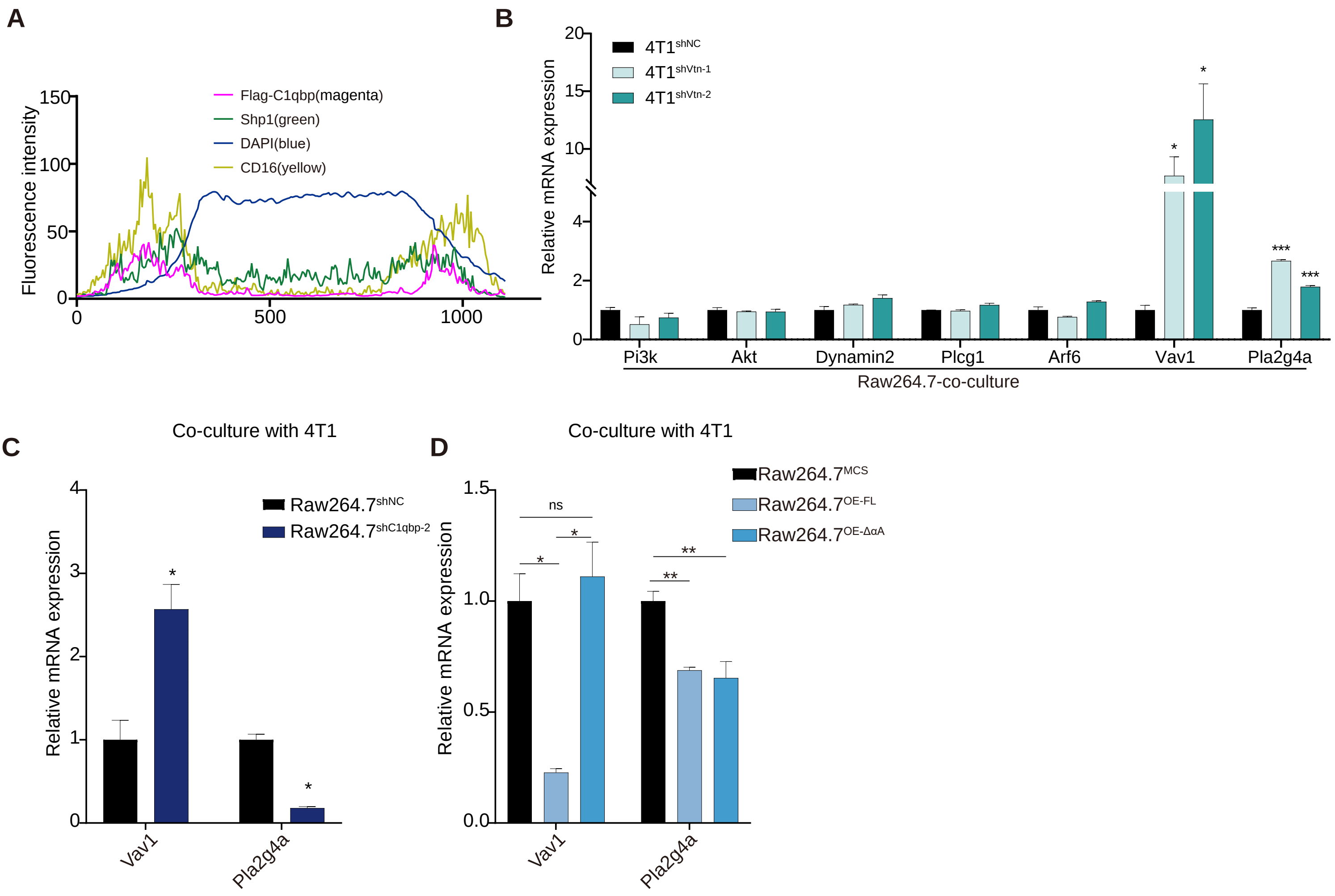

Fig. S8 The co-localization of CD16, C1qbp, and Shp1 and the effect of Vtn-C1qbp on the expression of molecules downstream of Syk

(A) Intensity profiles of all four fluorescent channels in Fig.6K obtained using ImageJ software, along a rectangular area (white dotted line) crossing the nucleus of a representative cell indicated on the overlay images. **(B)** qRT-PCR analysis of Syk downstream molecule in Raw264.7 cells stimulated with Vtn knockdown or control 4T1 cells for 48 h. **(C and D)** qRT-PCR analysis of Vav1 and Pla2g4a gene in control (Raw264.7^{shNC}) and C1qbp-knockdown Raw264.7 cells (Raw264.7$^{shC1qbp-2}$) (C); Full-length C1qbp (Raw264.7^{OE-FL}), mutant-C1qbp (Raw264.7$^{OE-\Delta\alpha A}$) overexpression and control Raw264.7 (Raw264.7^{MCS}) (D) co-culture with 4T1 cells for 48h.

Fig.S9

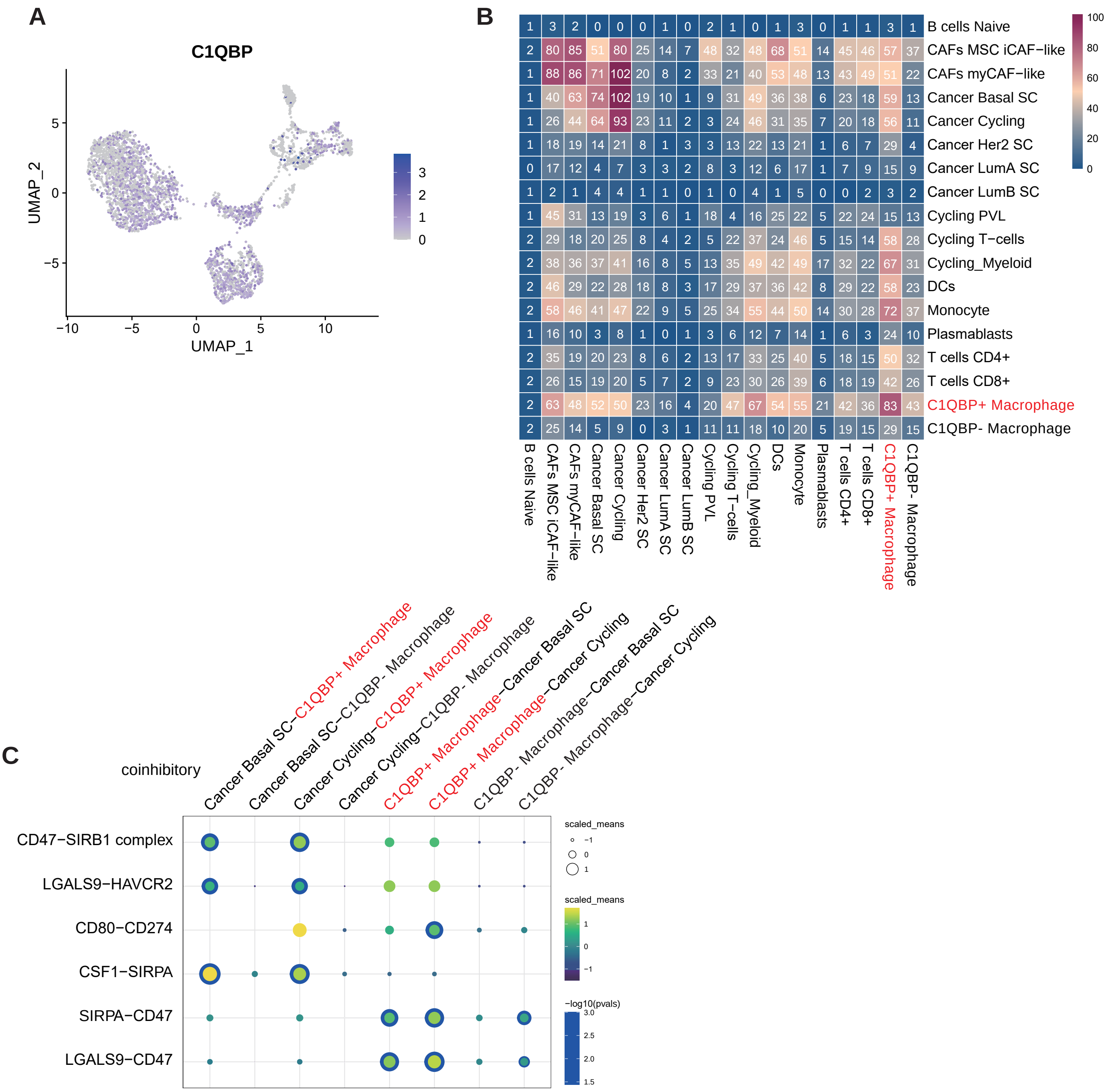

Fig. S9 C1qbp$^+$ macrophages have more inhibitory potential than C1qbp$^-$ macrophages in TNBC patients

(A) UMAP plot showing C1QBP expression levels on clusters of macrophages. **(B)** Heatmap showing the total number of interactions between cell types with CellPhoneDB analysis. **(C)** Dot plot showing the interaction strength for immunosuppressive ligand-receptor pairs on C1QBP$^+$ macrophage or C1QBP$^-$ macrophage with cancer cells with CellPhoneDB analysis. Dot size and color show the interaction strength levels, blue circle indicates p value.

Fig.S10

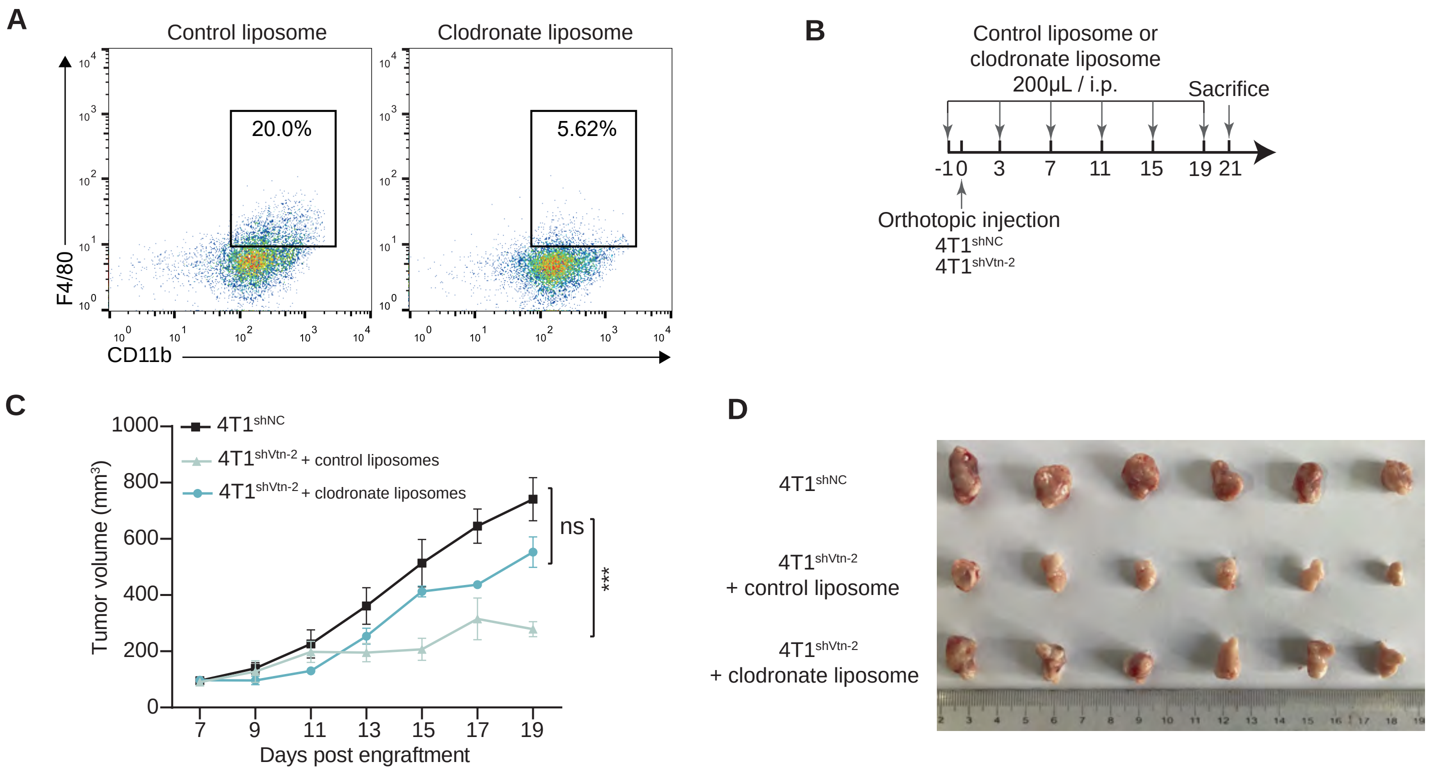

Fig. S10 Attenuation of anti-tumor activity of Vtn knockdown by macrophage depletion. **(A)** Macrophage depletion was confirmed by flow cytometry. **(B)** Schematic diagram of the mouse experiment (6 mice in each group). **(C and D)** Tumor volumes (C) and representative images (D) were measured (n = 6, mean ± SEM, ns not significant, *P < 0.05, **P < 0.01, ***P < 0.001).

Fig.S11

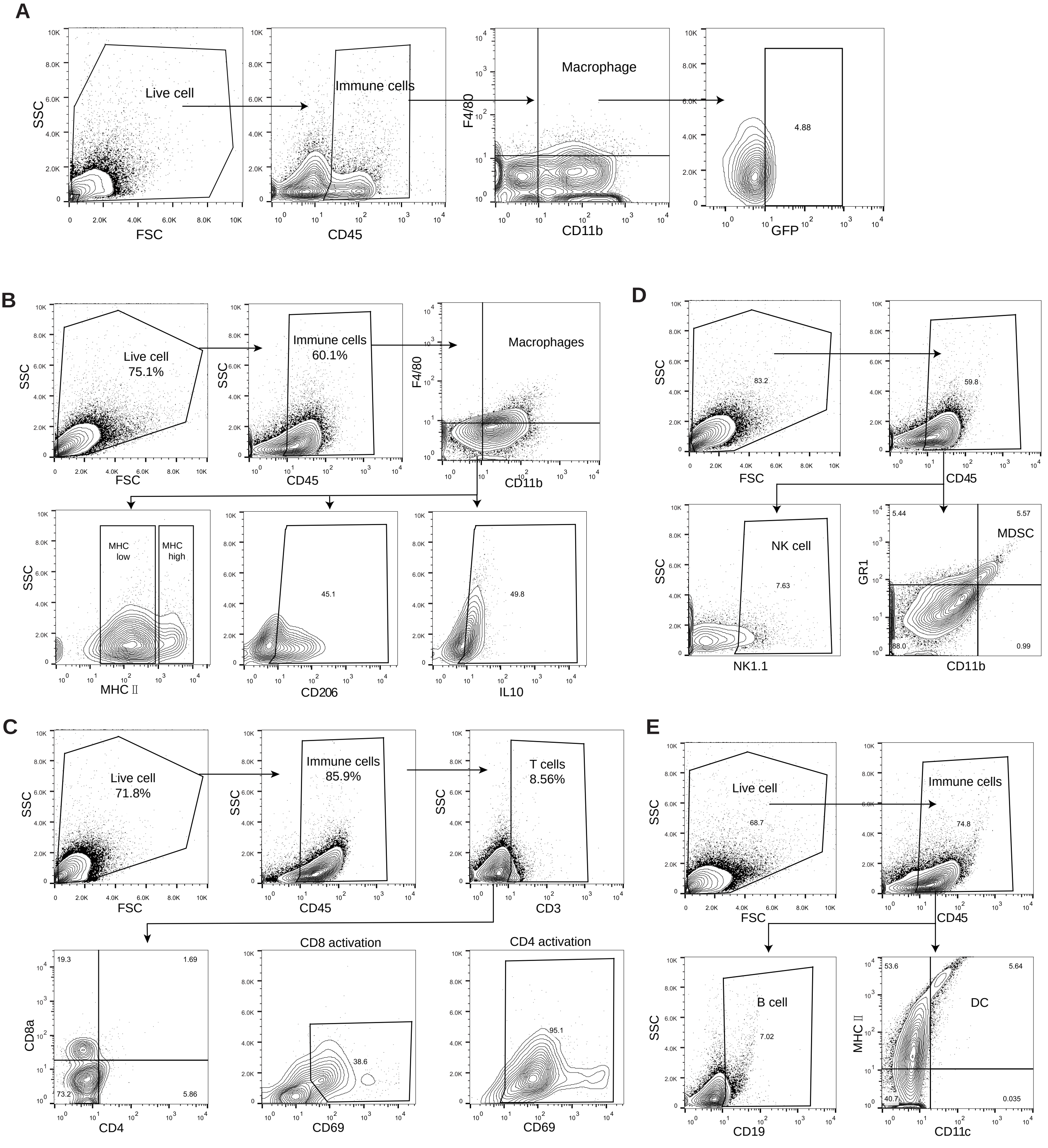
A
Live cell
Immune cells
Macrophage
4.88
SSC
FSC
CD45
F4/80
CD11b
GFP
B
Live cell
75.1%
Immune cells
60.1%
Macrophages
MHC low
MHC high
45.1
49.8
MHC Ⅱ
CD206
IL10
C
Live cell
71.8%
Immune cells
85.9%
T cells
8.56%
19.3
1.69
73.2
5.86
CD8a
CD4
CD8 activation
38.6
CD4 activation
95.1
CD69
CD3
D
83.2
59.8
NK cell
7.63
NK1.1
5.44
5.57
MDSC
88.0
0.99
GR1
E
Live cell
68.7
Immune cells
74.8
B cell
7.02
CD19
53.6
5.64
DC
40.7
0.035
CD11c

Fig. S11 Gating strategy for flow cytometry

(A) Gating strategy for in vivo TAM phagocytosis of 4T1 cells; TAM phagocytosis is assessed as the frequency of $CD45^+CD11b^+F4/80^+GFP^+$ events out of total $CD45^+CD11b^+F4/80^+$events.

(B to E) The figure depicts the gating strategies for identifying $MHCII^+$, $CD206^+$, $IL10^+$ macrophages (B), $CD4^+T/CD8^+T$ cells and the activated T cells (C)**,** NK cells, MDSC (D) B cells and DC cells (E) present in the tumor of tumor-bearing mice.

Supplementary Tables

Table S1: the antibodies used in article

Antibodies	SOURCE	IDENTIFIER	Application
Anti-Vitronectin antibody	Proteintech	15833-1-AP	WB/IHC/IF/FACS
Anti-C1qbp antibody	Proteintech	24474-1-AP	WB/IHC/IF/FACS/IP
Anti-Flag antibody	Proteintech	66008-4-Ig	WB/IP
Anti-Myc tag antibody	Proteintech	16286-1-AP	WB/IP
Anti-Flag antibody	Proteintech	20543-1-AP	WB/IP
Anti-Rabbit IgG, Light Chain	Proteintech	SA00001-7L	WB
Anti-Rabbit IgG, heavy Chain	Proteintech	HRP-66467	WB
Anti-β-actin antibody	Proteintech	81115-1-RR	WB
HRP-conjugated Affinipure Goat Anti-Rabbit IgG(H+L)	Proteintech	SA00001-2	WB
Anti-Syk antibody	CST	13198	WB
Anti-P-Syk antibody	CST	2717	WB
Anti-CD45 antibody	Santa Cruz	sc-1178	IF
CD68	Abcam	ab125212	IHC
F4/80-FITC	Biolegend	123107	FACS
I-A/I-E-PE	Biolegend	107607	FACS
CD45-PerCP	Biolegend	103130	FACS
CD11b-APC	Biolegend	101212	FACS
IFN-γ-PE	Biolegend	505807	FACS

CD206-PE	Biolegend	141705	FACS
IL-10-PE	Biolegend	505007	FACS
CD4-FITC	Biolegend	130308	FACS
CD8a-FITC	Biolegend	100803	FACS
CD25-FITC	Biolegend	101907	FACS
CD11c-FITC	Biolegend	117305	FACS
CD11b-FITC	Biolegend	101205	FACS
CD3-FITC	Biolegend	100203	FACS
CD8a-PE	Biolegend	162303	FACS
CD69-PE	Biolegend	104507	FACS
CD3-PE	Biolegend	100205	FACS
NK1.1-PE	Biolegend	156503	FACS
CD4-PercP	Biolegend	100432	FACS
CD3-APC	Biolegend	100236	FACS
FOXP3-Alexa Fluor 647	Biolegend	126408	FACS
CD19-APC	Biolegend	152409	FACS
GR1-APC	Biolegend	108411	FACS

Table S2: The sequences of primers for RT-qPCR

Genes	Primer
Mouse-CD206	F: GCTTGTAGGAAGGAGGGT
	R: TCCAGGAAGCCATTTAGT
Mouse-Arg1	F: CTCCAAGCCAAAGTCCTTAGAG
	R: AGGAGCTGTCATTAGGGACATC
Mouse-Inos	F: GTTCTCAGCCCAACAATACAAGA
	R: GTGGACGGGTCGATGTCAC
Mouse-β-actin	F: GGTCCACACCCGCCACCAG
	R: CACATGCCGGAGCCGTTGTC
Mouse-Vtn	F: CCCCTGAGGCCCTTTTTCATA
	R: CAAAGCTCGTCACACTGACA
Mouse-C1qbp	F: AAGATCCAGAAACACAAGTCCCT
	R: CCTCCTCACCATCAAATGTTGG
